# The SARS-CoV-2 Conserved Macrodomain Is a Mono-ADP-Ribosylhydrolase

**DOI:** 10.1128/JVI.01969-20

**Published:** 2021-01-13

**Authors:** Yousef M. O. Alhammad, Maithri M. Kashipathy, Anuradha Roy, Jean-Philippe Gagné, Peter McDonald, Philip Gao, Louis Nonfoux, Kevin P. Battaile, David K. Johnson, Erik D. Holmstrom, Guy G. Poirier, Scott Lovell, Anthony R. Fehr

**Affiliations:** a Department of Molecular Biosciences, University of Kansas, Lawrence, Kansas, USA; b Protein Structure Laboratory, University of Kansas, Lawrence, Kansas, USA; c High Throughput Screening Laboratory, University of Kansas, Lawrence, Kansas, USA; d Department of Molecular Biology, Medical Biochemistry and Pathology, Laval University Cancer Research Center, Québec City, Quebec, Canada; e Oncology Division, CHU de Québec Research Center, Québec City, Quebec, Canada; f Protein Production Group, University of Kansas, Lawrence, Kansas, USA; g NYX, New York Structural Biology Center, Upton, New York, USA; h Molecular Graphics and Modeling Laboratory and the Computational Chemical Biology Core, University of Kansas, Lawrence, Kansas, USA; Loyola University Chicago

**Keywords:** coronavirus, SARS-CoV-2, macrodomain, ADP-ribose, poly-ADP-ribose, mono-ADP-ribose

## Abstract

Severe acute respiratory syndrome coronavirus 2 (SARS-CoV-2) and other SARS-related CoVs encode 3 tandem macrodomains within nonstructural protein 3 (nsp3). The first macrodomain, Mac1, is conserved throughout CoVs and binds to and hydrolyzes mono-ADP-ribose (MAR) from target proteins. Mac1 likely counters host-mediated antiviral ADP-ribosylation, a posttranslational modification that is part of the host response to viral infections. Mac1 is essential for pathogenesis in multiple animal models of CoV infection, implicating it as a virulence factor and potential therapeutic target. Here, we report the crystal structure of SARS-CoV-2 Mac1 in complex with ADP-ribose. SARS-CoV-2, SARS-CoV, and Middle East respiratory syndrome coronavirus (MERS-CoV) Mac1 domains exhibit similar structural folds, and all 3 proteins bound to ADP-ribose with affinities in the low micromolar range. Importantly, using ADP-ribose-detecting binding reagents in both a gel-based assay and novel enzyme-linked immunosorbent assays (ELISAs), we demonstrated de-MARylating activity for all 3 CoV Mac1 proteins, with the SARS-CoV-2 Mac1 protein leading to a more rapid loss of substrate than the others. In addition, none of these enzymes could hydrolyze poly-ADP-ribose. We conclude that the SARS-CoV-2 and other CoV Mac1 proteins are MAR-hydrolases with similar functions, indicating that compounds targeting CoV Mac1 proteins may have broad anti-CoV activity.

**IMPORTANCE** SARS-CoV-2 has recently emerged into the human population and has led to a worldwide pandemic of COVID-19 that has caused more than 1.2 million deaths worldwide. With no currently approved treatments, novel therapeutic strategies are desperately needed. All coronaviruses encode a highly conserved macrodomain (Mac1) that binds to and removes ADP-ribose adducts from proteins in a dynamic posttranslational process that is increasingly being recognized as an important factor that regulates viral infection. The macrodomain is essential for CoV pathogenesis and may be a novel therapeutic target. Thus, understanding its biochemistry and enzyme activity are critical first steps for these efforts. Here, we report the crystal structure of SARS-CoV-2 Mac1 in complex with ADP-ribose and describe its ADP-ribose binding and hydrolysis activities in direct comparison to those of SARS-CoV and MERS-CoV Mac1 proteins. These results are an important first step for the design and testing of potential therapies targeting this unique protein domain.

## Abstract (precis)

SARS-CoV-2 has recently emerged into the human population and has led to a worldwide pandemic of COVID-19 that has caused more than 1.2 million deaths worldwide. With no currently approved treatments, novel therapeutic strategies are desperately needed.

## INTRODUCTION

The recently emerged pandemic outbreak of coronavirus disease 2019 (COVID-19) is caused by a novel coronavirus named severe acute respiratory syndrome coronavirus 2 (SARS-CoV-2) (1, 2). As of 2 November 2020, this virus has been responsible for <46 million cases of COVID-19 and >1.2 million deaths worldwide. SARS-CoV-2 is a member of the subgenus *Sarbecovirus* of the genus *Betacoronavirus* (β-CoVs) with overall high sequence similarity with other severe acute respiratory syndrome-related coronaviruses, including SARS-CoV. While most of the genome is >80% similar to SARS-CoV, there are regions where amino acid conservation is significantly lower. As expected, the most divergent proteins in the SARS-CoV-2 genome from SARS-CoV include the spike glycoprotein and several accessory proteins, including 8a (absent), 8b (extended), and 3b (truncated). However, somewhat unexpectedly, several nonstructural proteins also show significant divergence from SARS-CoV, including nonstructural proteins 3, 4, and 7, which could affect the biology of SARS-CoV-2 (3, 4).

**FIG 1:**
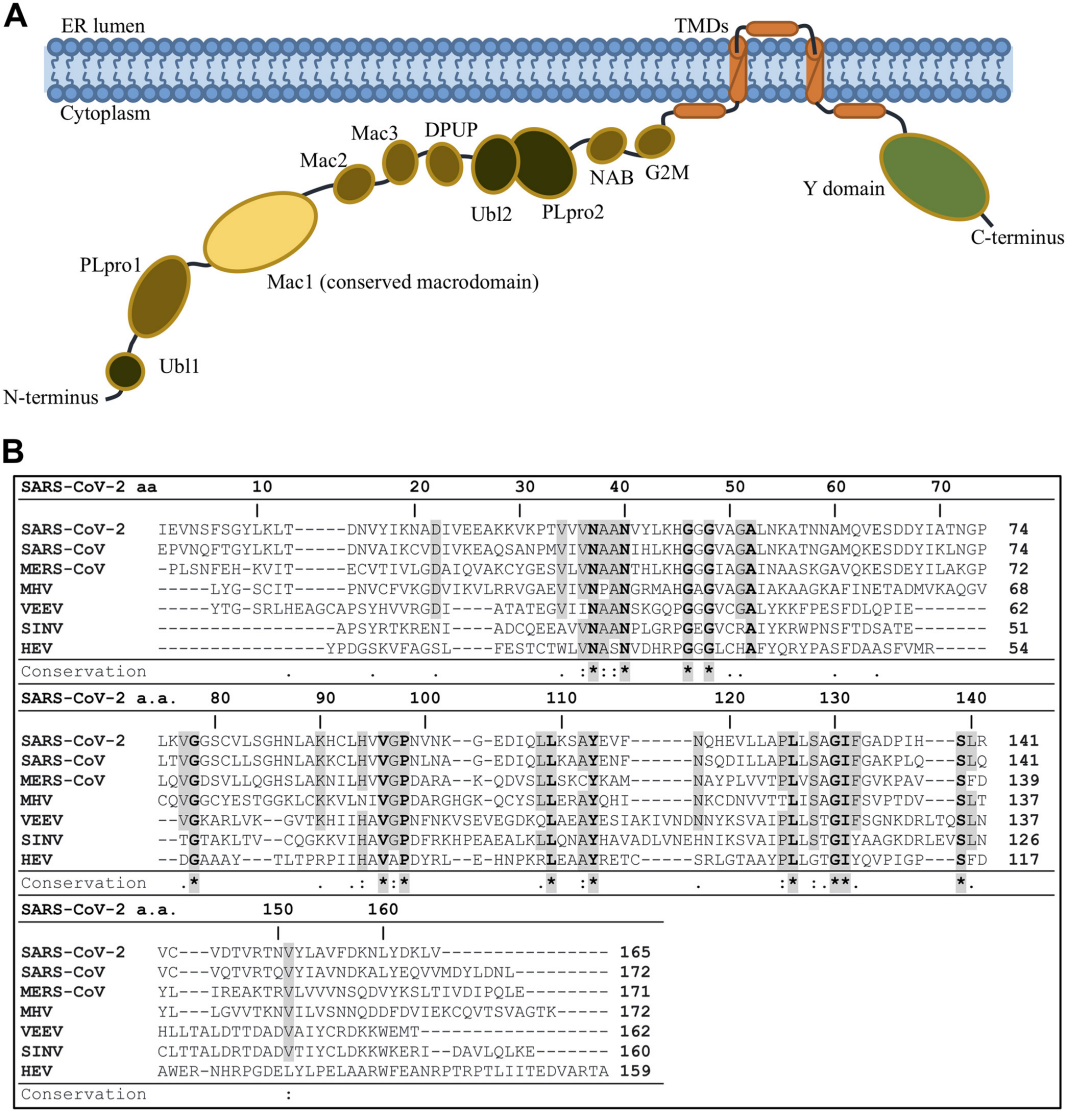
The SARS-CoV-2 Mac1 is a small domain within nsp3 and is highly conserved between other human CoV Mac1 protein domains. (A) Cartoon schematic of the SARS-CoV-2 nonstructural protein 3. The conserved macrodomain (Mac1) is in yellow. (B) Sequence alignment of Mac1 from CoVs; SARS-CoV-2, SARS-CoV, MERS-CoV, and mouse hepatitis virus (MHV), from the alphaviruses Venezuelan equine encephalitis virus (VEEV) and Sindbis virus (SINV), and from hepatitis E virus (HEV). Sequences were aligned using the ClustalW method from Clustal Omega online tool with manual adjustment. Identical residues are boldface, shaded in gray, and marked with asterisks; semiconserved residues are shaded in gray and marked with two dots (one change among all viruses) or one dot (two changes or conserved within the CoV family).

Coronaviruses encode 16 nonstructural proteins that are processed from two polyproteins, 1a and 1ab (pp1a and pp1ab) (5). The largest nonstructural protein is nonstructural protein 3 (nsp3), which contains multiple modular protein domains. These domains in SARS-CoV-2 diverge in amino acid sequence from SARS-CoV as much as 30%. The SARS-CoV-2 nsp3 includes three tandem macrodomains (Mac1, Mac2, and Mac3) (Fig. 1A) (3). The individual macrodomains of SARS-CoV-2 show similar, if not more, amino acid divergence compared to the other domains of nsp3 and more divergence than all nonstructural proteins except nsp4 and nsp7. Mac1 diverges 28% from SARS-CoV and 59% from Middle East respiratory syndrome coronavirus (MERS-CoV), while Mac2 and Mac3 diverge 24% from SARS-CoV. It is feasible that these significant sequence differences could impact the unique biology of SARS-CoV-2. However, macrodomains have a highly conserved structure, and thus, sequence divergence may have little impact on their overall function. Mac1 is present in all CoVs, unlike Mac2 and Mac3, and early structural and biochemical data demonstrated that it contains a conserved three-layered α/β/α fold and binds to mono-ADP-ribose (MAR) and other related molecules (6–10). This is unlike Mac2 and Mac3, which fail to bind ADP-ribose and instead appear to bind to nucleic acids (11, 12). ADP-ribose is buried in a hydrophobic cleft of Mac1, where the ADP-ribose binds to several highly conserved residues, such as an aspartic acid at position 1022 (D1022) of SARS-CoV pp1a (D22 of SARS-CoV and SARS-CoV-2 Mac1) and asparagine at position 1040 of pp1a (N1040) (N40 of SARS-CoV and SARS-CoV-2 Mac1) (Fig. 1B) (6). Mac1 homologs are also found in alphaviruses, hepatitis E virus, and rubella virus, and structural analysis of these macrodomains has demonstrated that they are very similar to CoV Mac1 (13, 14). All are members of the larger MacroD-type macrodomain family, which includes human macrodomains Mdo1 and Mdo2 (15).

The CoV Mac1 was originally named ADP-ribose-1″-phosphatase (ADRP) based on data demonstrating that it could remove the phosphate group from ADP-ribose-1″-phosphate (6–8). However, the activity was rather modest, and it was unclear why this would impact a virus infection. More recently it has been demonstrated that CoV Mac1 can hydrolyze the bond between amino acid chains and ADP-ribose molecules (16–18), indicating that it can reverse protein ADP-ribosylation (6, 8). ADP-ribosylation is a posttranslational modification catalyzed by ADP-ribosyltransferases [ARTs; also known as poly(ADP-ribose) polymerases (PARPs)] through transferring an ADP-ribose moiety from NAD^+^ onto target proteins (19). The ADP-ribose is transferred as a single unit of MAR, or single units of MAR are transferred consecutively to form a PAR chain. Several Mac1 proteins have been shown to hydrolyze MAR but have minimal activity for PAR (16, 17). Several MARylating PARPs are induced by interferon (IFN) and are known to inhibit virus replication, implicating MARylation in the host response to infection (20).

Several reports have addressed the role of Mac1 in the replication and pathogenesis of CoVs, mostly using the mutation of a highly conserved asparagine to alanine (N41A-SARS-CoV). This mutation abolished the MAR-hydrolase activity of SARS-CoV Mac1 (18). This mutation has minimal effects on CoV replication in transformed cells but reduces viral load, leads to enhanced IFN production, and strongly attenuates both murine hepatitis virus (MHV) and SARS-CoV in mouse models of infection (7, 18, 21, 22). MHV Mac1 was also required for efficient replication in primary macrophages, which could be partially rescued by the PARP inhibitors XAV-939 and 3-AB or small interfering RNA (siRNA) knockdown of PARP12 or PARP14 (23). These data suggest that Mac1’s likely function is to counter PARP-mediated antiviral ADP-ribosylation (24). Mutations in the alphavirus and hepatitis E virus (HEV) macrodomain also have substantial phenotypic effects on virus replication and pathogenesis (16, 25–28). As viral macrodomains are clearly important virulence factors, they are considered potential targets for antiviral therapeutics (24).

Based on the close structural similarities between viral macrodomains, we hypothesized that SARS-CoV-2 Mac1 has binding and hydrolysis activities similar to those of other CoV Mac1 enzymes. In this study, we determined the crystal structure of the SARS-CoV-2 Mac1 protein bound to ADP-ribose. Binding to and hydrolysis of MAR were tested and directly compared to those of a human macrodomain (Mdo2) and the SARS-CoV and MERS-CoV Mac1 proteins by several *in vitro* assays. All CoV Mac1 proteins bound to MAR and could remove MAR from a protein substrate. However, the initial rate associated with the loss of substrate was highest for the SARS-CoV-2 Mac1 protein, especially under multiturnover conditions. In addition, none of these enzymes could remove PAR from a protein substrate. These results indicate that Mac1 protein domains likely have similar functions and will be instrumental in the design and testing of novel therapeutic agents targeting the CoV Mac1 protein domain.

## RESULTS

### Structure of the SARS-CoV-2 Mac1 complexed with ADP-ribose.

To create recombinant SARS-CoV-2 Mac1 for structure determination and enzyme assays, nucleotides 3348 to 3872 of SARS-CoV-2 isolate Wuhan-hu-1 (accession number NC_045512), representing amino acids I1023 to K1197 of pp1a, were cloned into a bacterial expression vector containing an N-terminal 6-His tag and TEV (tobacco etch virus) protease cleavage site. We obtained large amounts (>100 mg) of purified recombinant protein. A small amount of this protein was digested by the TEV protease to obtain protein devoid of any extra tags for crystallization and used to obtain crystals from which the structure was determined. Our crystallization experiments resulted in the same crystal form (needle clusters) from several conditions, but only when ADP-ribose was added to the protein. This represents an additional crystal form (*P*2_1_) among the recently determined SARS-CoV-2 macrodomain structures (29, 30).

**FIG 2:**
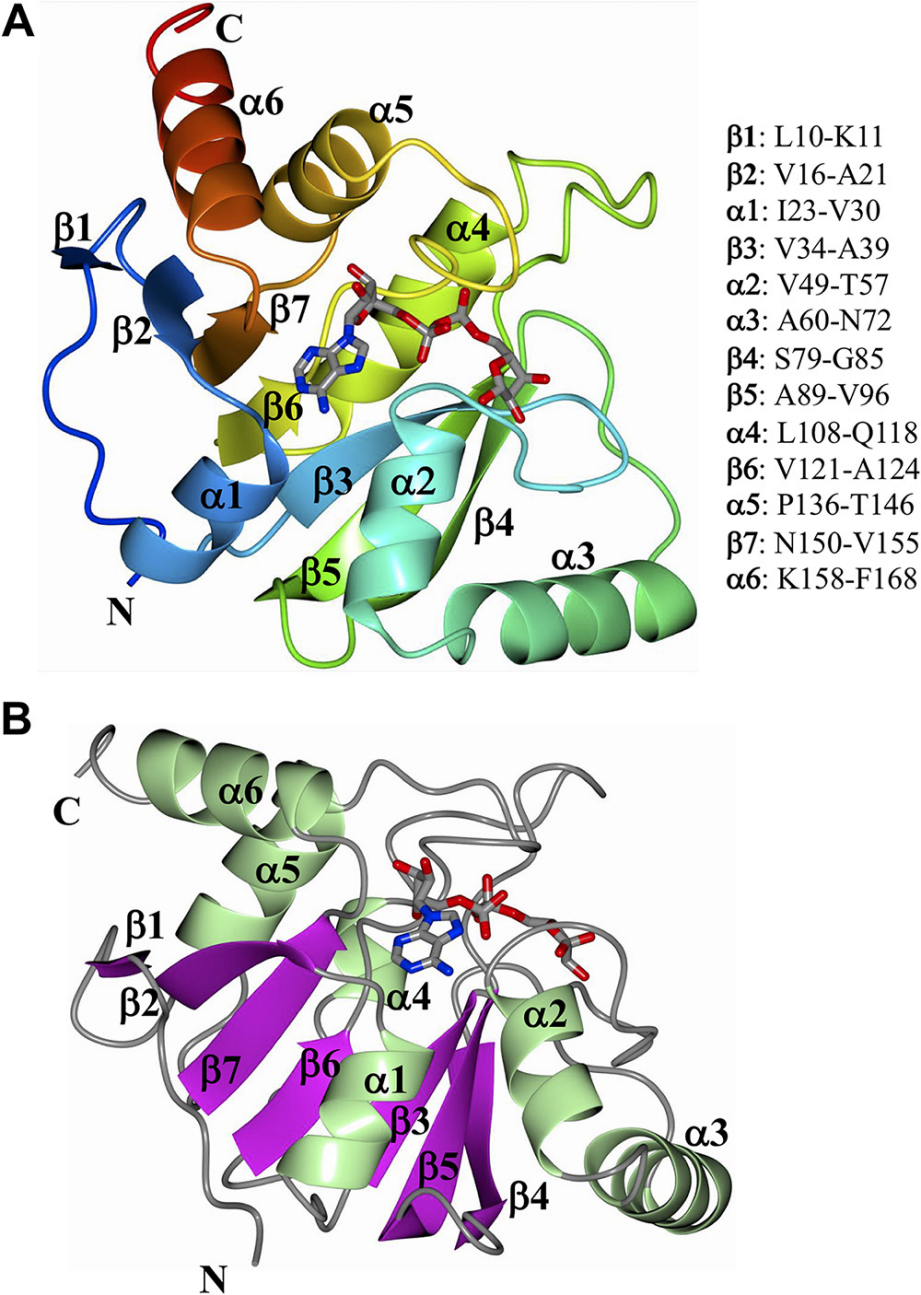
Structure of SARS-CoV-2 Mac1 complexed with ADP-ribose. (A) The structure was rendered as ribbons and colored using the visible spectrum from the N terminus (blue) to the C terminus (red). (B) The structure was colored by secondary structure showing sheets (magenta) and helices (green). The ADP-ribose is rendered as gray cylinders, with oxygens and nitrogens colored red and blue, respectively.

The structure of SARS-CoV-2 Mac1 complexed with ADP-ribose was obtained using X-ray diffraction data to 2.2 Å resolution and contained four molecules in the asymmetric unit that were nearly identical (Table 1). The polypeptide chains could be traced from V3-M171 for subunits A/C and V3-K172 for subunits B/D. Superposition of subunits B/D onto subunit A (169 residues aligned) yielded root mean square deviations (RMSD) of 0.17 Å, 0.17 Å, and 0.18 Å, respectively, between Cα atoms. As such, subunit A was used for the majority of the structure analysis described herein. The SARS-CoV-2 Mac1 protein adopted a fold consistent with the MacroD subfamily of macrodomains that contains a core composed of a mixed arrangement of 7 β-sheets (parallel and antiparallel) that are flanked by 6 α-helices (Fig. 2A and B).

**TABLE 1:** Crystallographic data for SARS-CoV-2 Mac1

Parameter	SARS-CoV-2 Mac1^*a*^
Data collection	
Unit-cell parameters (Å, °)	*a* = 59.72, *b* = 83.17, *c* = 84.24, β = 94.4
Space group	*P*2_1_
Resolution (Å)	48.41–2.20 (2.27–2.20)
Wavelength (Å)	1.0000
Temperature (K)	100
Observed reflections	144,767
Unique reflections	41,586
<*I*/σ(*I*)>	7.3 (1.9)
Completeness (%)	99.4 (99.7)
Multiplicity	3.5 (3.4)
*R*_merge_ (%)^*b*^	13.0 (67.0)
*R*_meas_ (%)^*c*^	15.4 (79.2)
*R*_pim_ (%)^*d*^	8.2 (41.8)
CC_1/2_^*e*^	0.994 (0.849)
Refinement	
Resolution (Å)	42.00–2.20
Reflections (working/test)	39,474/1,966
*R*_factor_/*R*_free_ (%)^*f*^	19.9/25.2
No. of atoms (protein/ligand/water)	4,930/144/358
Model quality	
RMSD	
Bond lengths (Å)	0.011
Bond angles (°)	1.144
Mean *B* factor (Å^2^)	
All atoms	28.1
Protein	27.9
Ligand	26.0
Water	30.9
Coordinate error (maximum likelihood) (Å)	0.31
Ramachandran plot	
Most favored (%)	97.3
Additionally allowed (%)	2.4

a Values in parentheses are for the highest-resolution shell.

b *R*_merge_ = Σ*_hkl_*Σ*_I_* |*I_i_*(*hkl*) − <*I*(*hkl*)>|/Σ*_hkl_*Σ*_I_ I_i_*(*hkl*), where *I_i_*(*hkl*) is the intensity measured for the *i*th reflection and <*I*(*hkl*)> is the average intensity of all reflections with indices *hkl*.

c *R*_meas_ = redundancy-independent (multiplicity-weighted) *R*_merge_ (47, 54).

d *R*_pim_ = precision-indicating (multiplicity-weighted) *R*_merge_ (55, 56).

e CC_1/2_ is the correlation coefficient of the mean intensities between two random half-sets of data (57, 58).

f *R*_factor_ = Σ*_hkl_* ‖*F*_obs_ (*hkl*) | − |*F*_calc_ (*hkl*) ‖/Σ*_hkl_* |*F*_obs_ (*hkl*)|; *R*_free_ is calculated in an identical manner using 5% of randomly selected reflections that were not included in the refinement.

**FIG 3:**
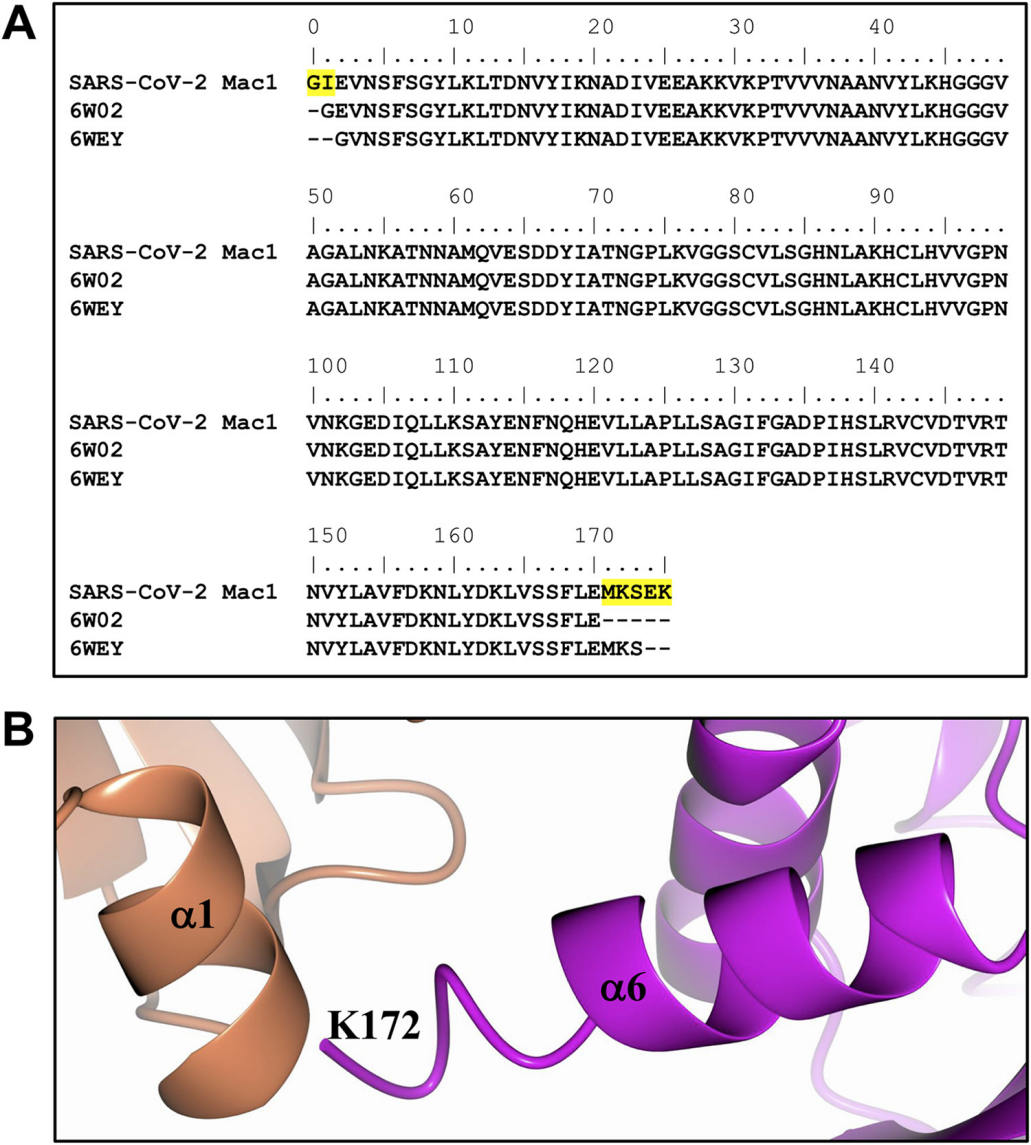
Extended residues at the C terminus of the SARS-CoV-2 Mac1 clash with symmetry-related molecules. (A) Comparison of the amino acid sequences of SARS-CoV-2 Mac1, 6W02, and 6WEY. (B) Superposition of SARS-CoV-2 Mac1 (magenta) subunit B onto subunit A of 6W02 reveals that the C terminus would clash with symmetry-related molecules (coral).

As mentioned above, apo crystals were never observed for our construct, though the apo structure has been solved by researchers at The Center for Structural Genomics of Infectious Diseases (PDB code 6WEN) (30) and the University of Wisconsin–Milwaukee (PDB code 6WEY) (31). Further analysis of the amino acid sequences used for expression and purification revealed that our construct had 5 additional residues at the C terminus (MKSEK) and differs slightly at the N terminus as well (GIE versus GE) relative to 6WEN. In addition, the sequence used to obtain the structure of 6WEY is slightly shorter than SARS-CoV-2 Mac1 at both the N- and C-terminal regions (Fig. 3A). To assess the effect of these additional residues on crystallization, chain B of the SARS-CoV-2 Mac1, which was traced to residue K172, was superimposed onto subunit A of the protein with PDB code 6W02 (30), a previously determined structure of ADP-ribose bound SARS-CoV-2 Mac1. Analysis of the crystal packing of 6W02 indicates that the additional residues at the C terminus would clash with symmetry-related molecules (Fig. 3B). This suggests that the presence of these extra residues at the C terminus likely prevented the generation of the more tightly packed crystal forms obtained for 6W02 and 6WEY, which diffracted to high resolution.

**FIG 4:**
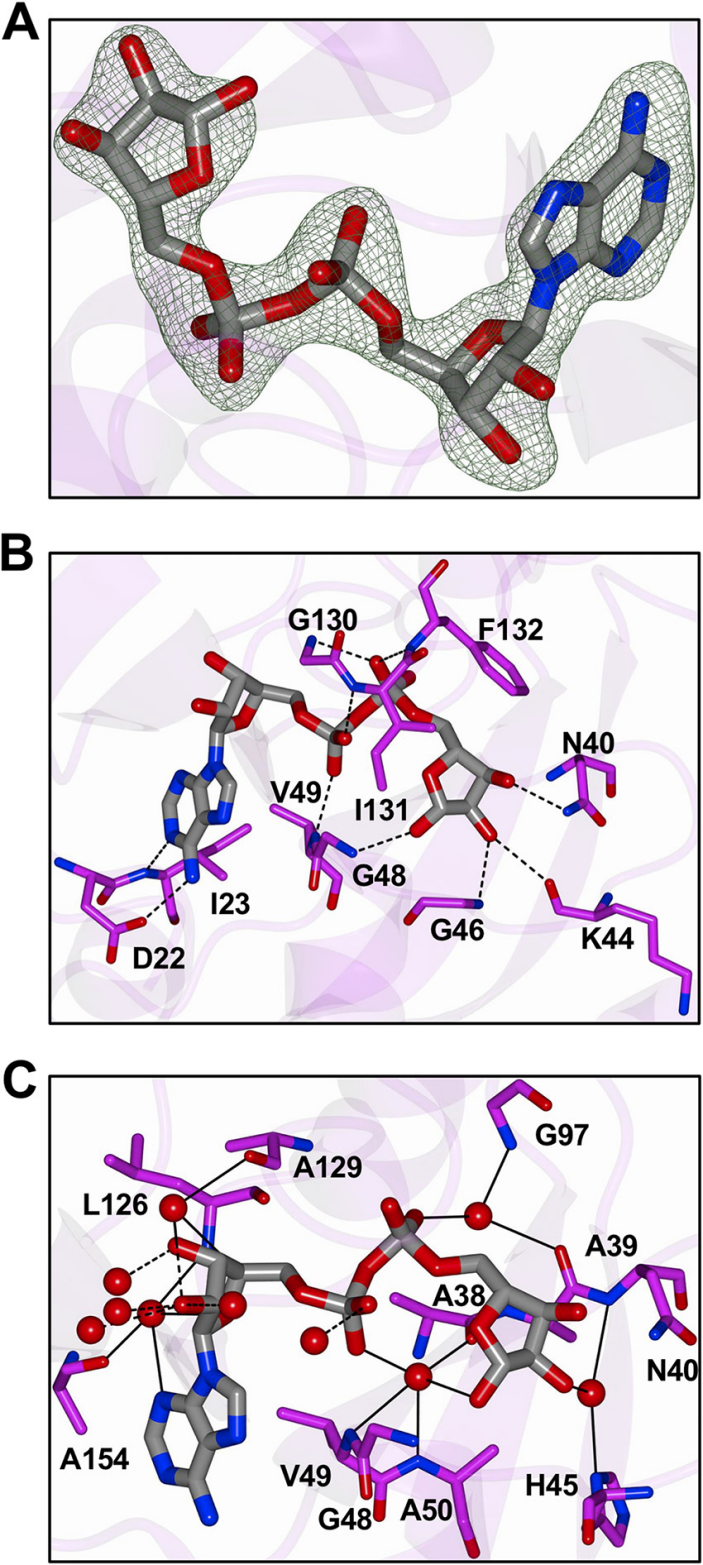
Binding mode of ADP-ribose in SARS-CoV-2 Mac1. (A) *F*_o_-*F*_c_ Polder omit map (green mesh) contoured at 3σ. (B) Hydrogen bond interactions (dashed lines) between ADP-ribose and amino acids. (C) Interactions with water molecules. Direct hydrogen bond interactions are represented by dashed lines, and water-mediated contacts to amino acids are represented by solid lines.

The ADP-ribose binding pocket contained large regions of positive electron density consistent with the docking of ADP-ribose (Fig. 4A). The adenine forms two hydrogen bonds with D22-I23, which makes up a small loop between β2 and the N-terminal half of α1. The side chain of D22 interacts with N6, while the backbone nitrogen atom of I23 interacts with N1, in a fashion very similar to that of the SARS-CoV macrodomain (6). This aspartic acid is known to be critical for ADP-ribose binding for alphavirus macrodomains (26, 27). A large number of contacts are made in the highly conserved loop between β3 and α2, which includes many highly conserved residues, including a GGG motif and N40, which is completely conserved in all enzymatically active macrodomains (32). N40 is positioned to make hydrogen bonds with the 3′ OH groups of the distal ribose, as well as a conserved water molecule (Fig. 4B and C). K44 and G46 also make hydrogen bonds with the 2′ OH of the distal ribose, and G48 makes contact with the 1′ OH and a water that resides near the catalytic site, while the backbone nitrogen atom of V49 hydrogen bonds with the α-phosphate. The other major interactions with ADP-ribose occur in another highly conserved region consisting of residues G130, I131, and F132, which are in the loop between β6 and α5 (Fig. 4B). The α-phosphate accepts a hydrogen bond from the nitrogen atom of I131, while the β-phosphate accepts hydrogen bonds from the backbone nitrogen atom of G130 and F132. The phenyl ring of F132 may make van der Waals interactions with the distal ribose to stabilize it, which may contribute to binding and hydrolysis (33). Loops β3-α2 and β6-α5 are connected by an isoleucine bridge that, following ADP-ribose binding, forms a narrow channel around the diphosphate which helps position the terminal ribose for water-mediated catalysis (6). Because there are only a few studies testing the activity of mutant forms of the macrodomain, is not exactly clear which of these residues are important for ADP-ribose binding, hydrolysis, or both. Additionally, a network of direct contacts of ADP-ribose to solvent along with water-mediated contacts to the protein are shown (Fig. 4C).

### Comparison of SARS-CoV-2 Mac1 with other CoV macrodomain structures.

**FIG 5:**
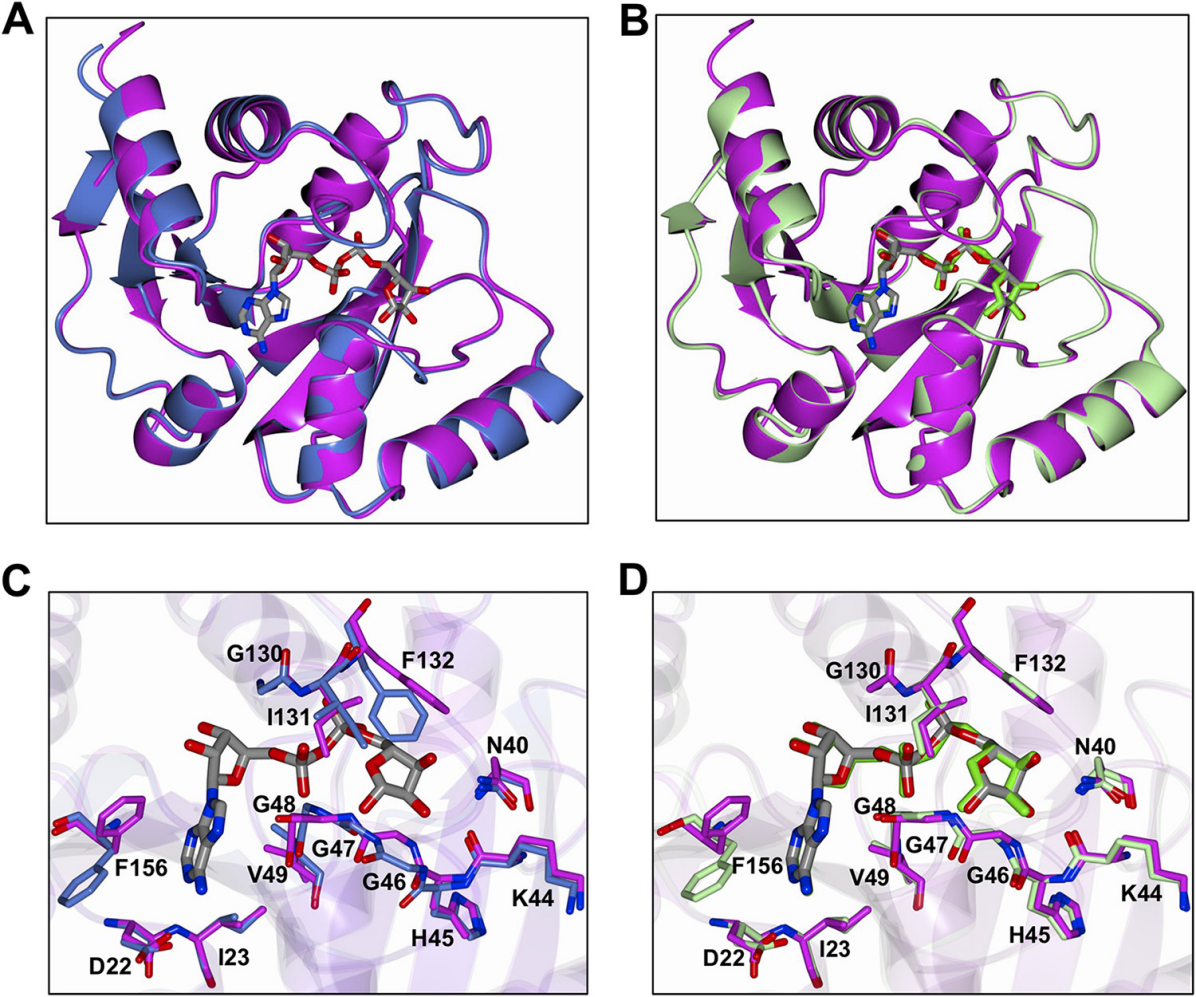
Comparison of the SARS-CoV-2 Mac1 protein with homologous structures. (A and B) Superposition of SARS-CoV-2 Mac1 (magenta) with other recently determined homologous structures. (A) SARS-CoV-2 Mac1 apo structure (6WEN); (B) SARS-CoV-2 Mac1 complexed with ADP-ribose (6W02). The ADP-ribose molecule is in gray for SARS-CoV-2 and is represented as green cylinders for 6W02 in panel B. (C and D) Comparison of the residues in the ADP-ribose binding site. (C) SARS-CoV-2 Mac1 apo structure (blue; 6WEN); (D) SARS-CoV-2 Mac1 complexed with ADP-ribose (green; 6W02). The ADP-ribose of SARS-CoV-2 is rendered as gray cylinders and is represented as green cylinders for 6W02 in panel B.

We next sought to compare the SARS-CoV-2 Mac1 to other deposited structures of this protein. Superposition with Apo (6WEN) and ADP-ribose complexed protein (6W02) yielded RMSD of 0.48 Å (168 residues) and 0.37 Å (165 residues), respectively, indicating a high degree of similarity (Fig. 5A and B). Comparison of the ADP-ribose binding site of SARS-CoV-2 Mac1 with that of the apo structure (6WEN) revealed minor conformational differences in order to accommodate ADP-ribose binding. The loop between β3 and α2 (H45-V49) undergoes a change in conformation, and the side chain of F132 is moved out of the ADP-ribose binding site (Fig. 5C). Our ADP-ribose-bound structure is nearly identical to 6W02, except for slight deviations in the β3-α2 loop and an altered conformation of F156, where the aryl ring of F156 is moved closer to the adenine ring (Fig. 5C and D). However, this is likely a result of crystal packing, as F156 adopts this conformation in each subunit and would likely clash with subunit residues related by either crystallographic or noncrystallographic symmetry.

**FIG 6:**
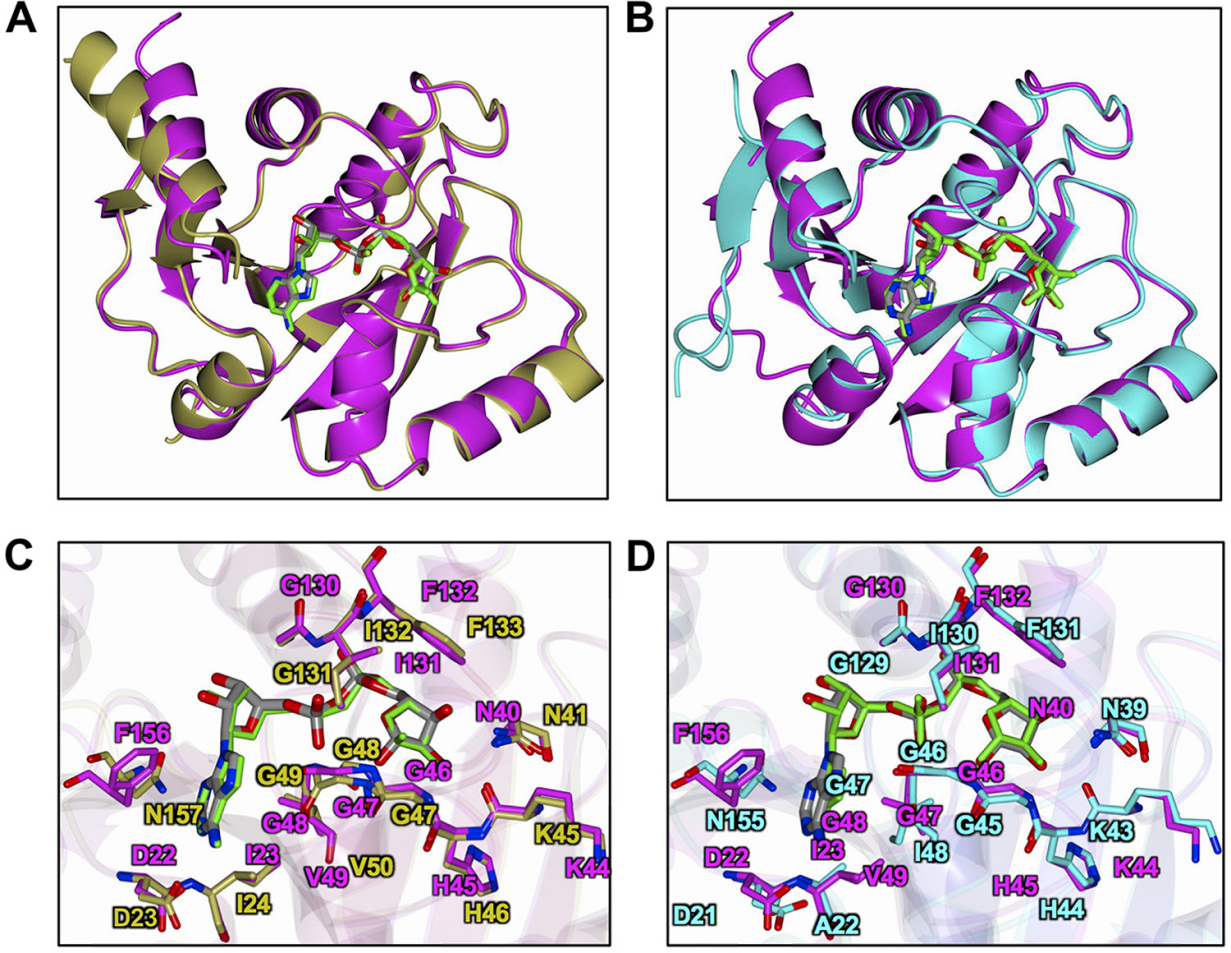
Structural comparison of the SARS-CoV-2 Mac1 protein with the SARS-CoV and MERS-CoV Mac1 proteins. (A and B) Superposition of SARS-CoV-2 macrodomain (magenta) with coronavirus macrodomain structures. (A) SARS-CoV Mac1 with ADP-ribose (gold) (2FAV); (B) MERS-CoV Mac1 with ADP-ribose (teal) (5HOL). (C and D) Superposition of SARS-CoV-2 Mac1 (magenta) with other coronavirus Mac1 structures highlighting the ADP-ribose binding site. (C) SARS-CoV (gold); (D) MERS-CoV (teal). The ADP-ribose molecules are colored gray for SARS-CoV-2 Mac1 (A to D) and are rendered as green cylinders for SARS-CoV Mac1 (A and C) and MERS-CoV Mac1 (B and D).

We next compared the ADP-ribose bound SARS-CoV-2 Mac1 structure with that of SARS-CoV (PDB code 2FAV) (6) and MERS-CoV (PDB code 5HOL) (34) Mac1 proteins (Fig. 6). Superposition yielded RMSD of 0.71 Å (166 residues) and 1.06 Å (161 residues) for 2FAV and 5HOL, respectively. Additionally, the ADP-ribose binding mode in the SARS-CoV and SARS-CoV-2 structures almost perfectly superimposed (Fig. 6A and C). The conserved aspartic acid residue (D22, SARS-CoV-2 Mac1) that binds to adenine is localized in a similar region in all 3 proteins, although there are slight differences in the rotamers about the Cβ-Cγ bond. The angles between the mean planes defined by the OD1, CG, and OD2 atoms relative to SARS-CoV-2 Mac1 are 23.1° and 46.5° for the SARS-CoV and MERS-CoV Mac1 structures, respectively. Another notable difference is that SARS-CoV and SARS-CoV-2 macrodomains have an isoleucine (I23) following this aspartic acid, while MERS-CoV has an alanine (A22) (Fig. 6C and D). Conversely, SARS-CoV-2 and SARS-CoV Mac1 have a valine instead of an isoleucine immediately following the GGG motif (V49/I48). From these structures, it appears that having two isoleucines in this location would clash and that the *Merbecovirus* and *Sarbecovirus* β-CoVs have evolved in unique ways to create space in this pocket (Fig. 6D and data not shown). Despite these small differences in local structure, the overall structure of CoV Mac1 domains remain remarkably conserved and indicates that they likely have similar biochemical activities and biological functions.

### SARS-CoV, SARS-CoV-2, and MERS-CoV bind to ADP-ribose with similar affinities.

**FIG 7:**
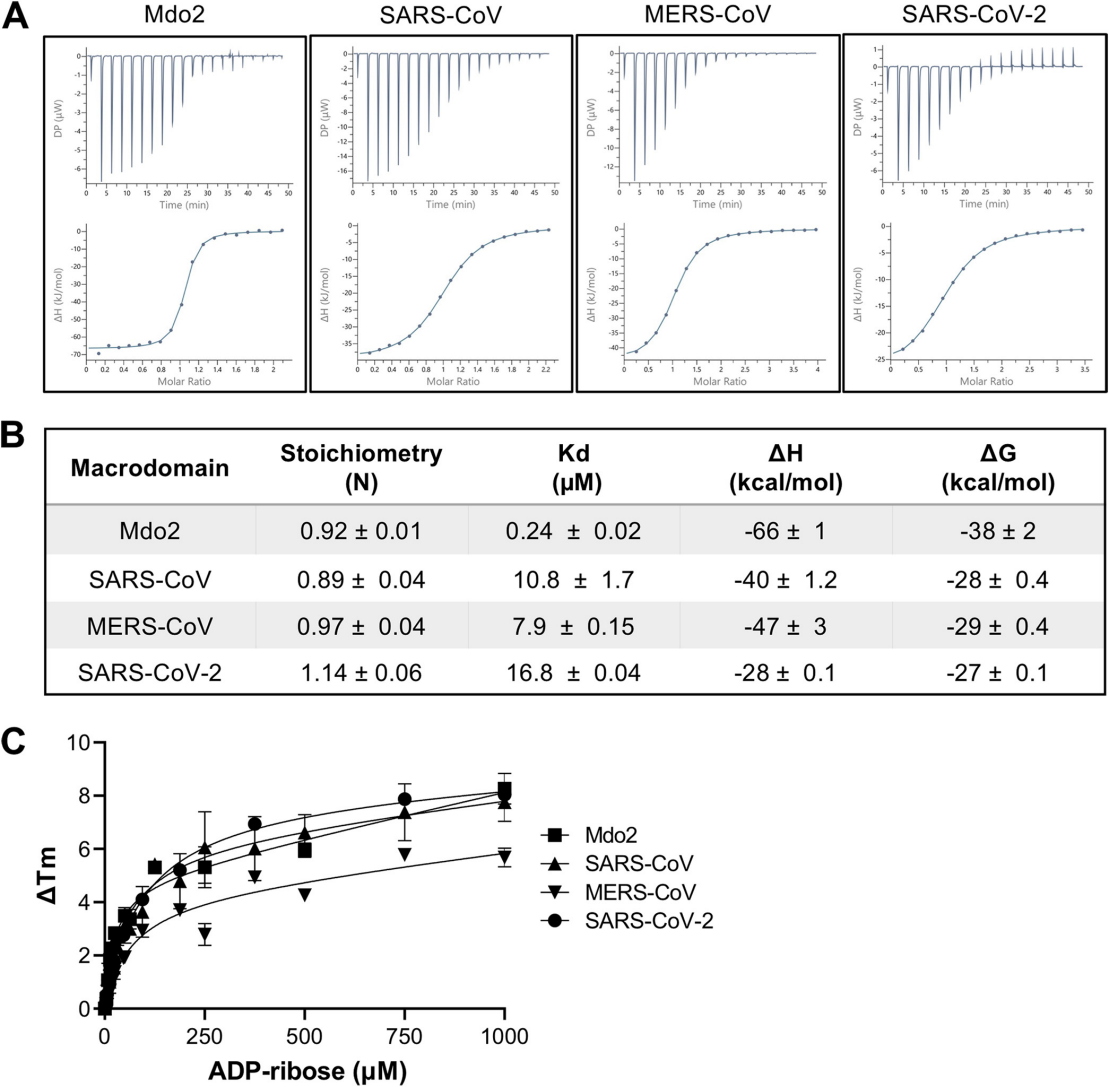
Human CoVs bind to ADP-ribose with similar affinity. (A and B) ADP-ribose binding of human Mdo2 and SARS-CoV, MERS-CoV, and SARS-CoV-2 Mac1 proteins by ITC. Images in panel A are from one experiment and are representative of at least 2 independent experiments. Data in panel B are combined averages from multiple independent experiments for each protein. Mdo2, *n* = 2; SARS-CoV, *n* = 5; MERS-CoV, *n* = 6; SARS-CoV-2, *n* = 2. (C) The macrodomain proteins (10 μM) were incubated with increasing concentrations of ADP-ribose and measured by DSF as described in Materials and Methods. Mdo2, *n* = 4; SARS-CoV, *n* = 6; MERS-CoV, *n* = 5; SARS-CoV-2, *n* = 3.

To determine if the CoV macrodomains had any noticeable differences in their ability to bind ADP-ribose, we performed isothermal titration calorimetry (ITC), which measures the energy released or absorbed during a binding reaction. Macrodomain proteins from human (Mdo2), SARS-CoV, MERS-CoV, and SARS-CoV-2 were purified and tested for their affinity to ADP-ribose. All CoV Mac1 proteins bound to ADP-ribose with low micromolar affinity (7 to 16 μM), while human Mdo2 bound with an affinity at least 30 times stronger (∼220 nM) (Fig. 7A and B). As a control, we tested the ability of the MERS-CoV macrodomain to bind to ATP and observed only minimal binding with millimolar affinity (data not shown). At higher concentrations, the SARS-CoV-2 macrodomain caused a slightly endothermic reaction, potentially the result of protein aggregation or a change in conformation (Fig. 7A). The MERS-CoV Mac1 had a greater affinity for ADP-ribose than SARS-CoV or SARS-CoV-2 Mac1 in the ITC assay (Fig. 7A and B); however, our results found the differences between these macrodomain proteins to be much smaller than previously reported (9). As an alternate method to confirm ADP-ribose binding, we conducted a thermal shift assay. All 4 macrodomains tested denatured at higher temperatures with the addition of ADP-ribose (Fig. 7C). We conclude that the *Merbecovirus* and *Sarbecovirus* Mac1 proteins bind to ADP-ribose with similar affinities.

### CoV macrodomains are MAR-hydrolases.

**FIG 8:**
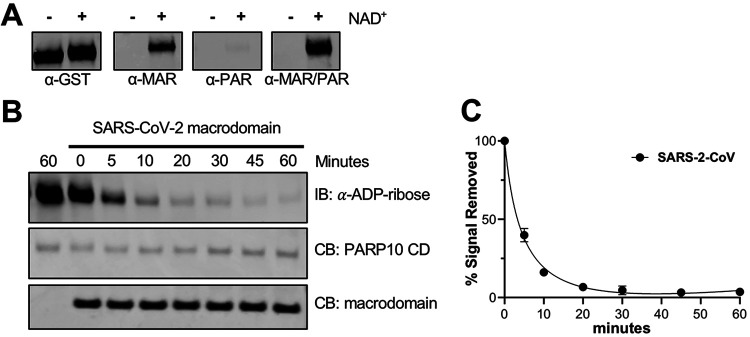
The SARS-CoV-2 Mac1 protein is a mono-ADP-ribosylhydrolase. (A) Affinity of ADP-ribose binding antibodies for ADP-ribosylated PARP10 CD. MARylated PARP10 and non-MARylated PARP10 CD were detected by immunoblotting (IB) with anti-GST (MA4-004; Invitrogen), anti-ADP-ribose binding reagents, and anti-MAR (MAB1076; MilliporeSigma), anti-PAR (MABC547; MilliporeSigma), and anti-MAR/PAR (MABE1075; MilliporeSigma) antibodies. (B) The SARS-CoV-2 macrodomain was incubated with MARylated PARP10 CD *in vitro* at equimolar ratios (1 μM) for the indicated times at 37°C. ADP-ribosylated PARP10 CD was detected by IB with anti-ADP-ribose binding reagent (MAB1076; MilliporeSigma). Total PARP10 CD and macrodomain protein levels were determined by Coomassie blue (CB) staining. The reaction with PARP10 CD incubated alone at 37°C was stopped at 0 or 60 min. (C) The level of de-MARylation was measured by quantifying band intensity using ImageJ software. Intensity values were plotted and fitsted to a nonlinear regression curve; error bars represent standard deviations. Results in panel A are from representative experiments of two independent experiments, and data in panel B are the combined results of the two independent experiments.

To examine the MAR-hydrolase activity of CoV Mac1, we first tested the viability of using ADP-ribose binding reagents to detect MARylated protein. Previously, the use of radiolabeled NAD^+^ has been the primary method for labeling MARylated protein (16, 17). To create a MARylated substrate, the catalytic domain of the PARP10 (glutathione *S*-transferase [GST]-PARP10 CD) protein was incubated with NAD^+^, leading to its automodification. PARP10 CD is a standard substrate that has been used extensively in the field to analyze the activity of macrodomains (16, 18, 26, 27). PARP10 is highly upregulated upon CoV infection (23, 35) and is known to primarily auto-MARylate on acidic residues, which are the targets of the MacroD2 class of macrodomains (27). We then tested a panel of anti-MAR, anti-PAR, or both anti-MAR and anti-PAR binding reagents/antibodies for the ability to detect MARylated PARP10 by immunoblotting. The anti-MAR and anti-MAR/PAR binding reagents, but not anti-PAR antibody, bound to MARylated PARP10 (Fig. 8A). Therefore, in this work, we utilized the anti-MAR binding reagent to detect MARylated PARP10.

We next tested the ability of SARS-CoV-2 Mac1 to remove ADP-ribose from MARylated PARP10. SARS-CoV-2 Mac1 and MARylated PARP10 were incubated at equimolar amounts of protein at 37°C, and the reaction was stopped at 5, 10, 20, 30, 45, or 60 min (Fig. 8B). As a control, MARylated PARP10 was incubated alone at 37°C and collected at similar time points (Fig. 8B). Each reaction had equivalent amounts of MARylated PARP10 and Mac1, which was confirmed by Coomassie blue staining (Fig. 8B). An immediate reduction of more than 50% band intensity was observed within 5 min, and the ADP-ribose modification was nearly completely removed by SARS-CoV-2 Mac1 within 30 min (Fig. 8B). The MARylated PAPR10 bands intensities were calculated, plotted, and fitted using nonlinear regression (Fig. 8C). This result indicates that the SARS-CoV-2 Mac1 protein is a mono-ADP-ribosylhydrolase enzyme.

**FIG 9:**
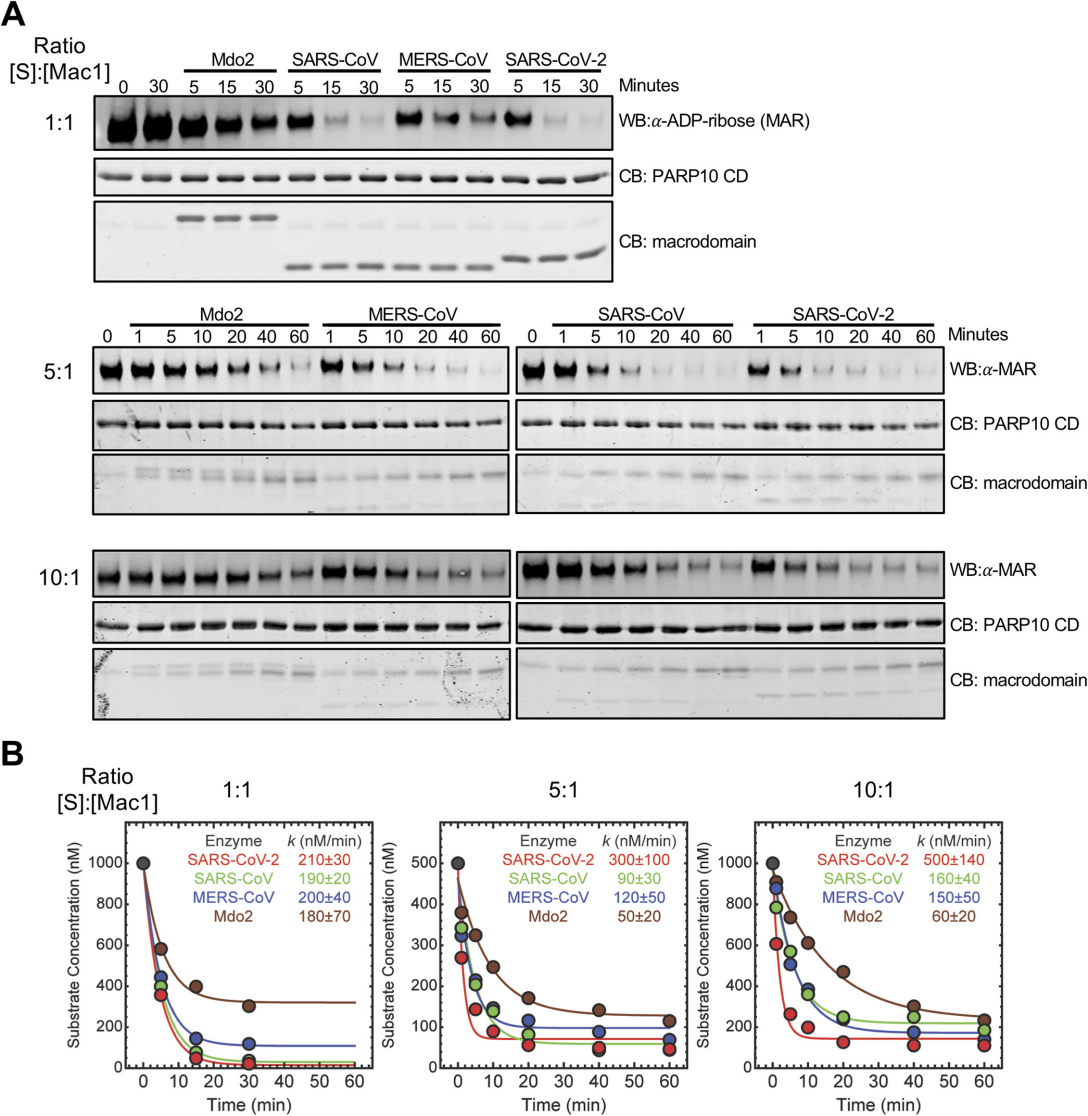
Comparison of the human Mdo2 and CoV Mac1 deMARylating activity. (A) The Mdo2, MERS-CoV, SARS-CoV, and SARS-CoV-2 macrodomains were incubated with MARylated PARP10 CD *in vitro* at [substrate]:[Mac1] ratios of 1:1 (1 μM), 5:1 (500 nM, 100 nM), and 10:1 (1 μM, 100 nM) for the indicated times at 37°C. ADP-ribosylated PARP10 CD was detected as described above, and total PARP10 CD and macrodomain protein levels were determined by Coomassie blue. (B) Time-dependent substrate concentrations were determined by quantifying band intensity using Image Studio software. The data were then analyzed using Mathematica 12, as described in Materials and Methods, to determine the initial rate (*k*) of substrate decay. Results in panel A are from representative experiments of three independent experiments, and data in panel B are the combined results of the three independent experiments.

Next, we compared the MAR-hydrolase activity of human Mdo2 and the Mac1 proteins from SARS-CoV-2, SARS-CoV, and MERS-CoV. Specifically, we monitored the time-dependent loss of substrate using immunoblotting (Fig. 9A) under equimolar (i.e., 1 μM Mac1:1 μM substrate) and multiple-turnover conditions (i.e., 0.5 μM substrate:0.1 μM Mac1 and 1.0 μM substrate:0.1 μM Mac1), with total protein amounts confirmed by Coomassie blue staining (Fig. 9A). The resulting substrate decay plots (Fig. 9B) were fitted using nonlinear regression to determine the initial rate (*k*) of substrate decay. Our results indicate that the three CoV Mac1 proteins give rise to similar, but not identical, values of *k* (Fig. 9B). The SARS-CoV-2 Mac1 protein has a greater *k* than the SARS-CoV or MERS-CoV Mac1 proteins, especially under multiple-turnover conditions, and all 3 viral macrodomains gave rise to a more rapid loss of substrate than the human Mdo2 enzyme (Fig. 9B). However, further enzymatic analyses of these proteins are warranted to more thoroughly understand their kinetics and binding affinities associated with various MARylated substrates.

### CoV Mac1 proteins do not hydrolyze PAR.

**FIG 10:**
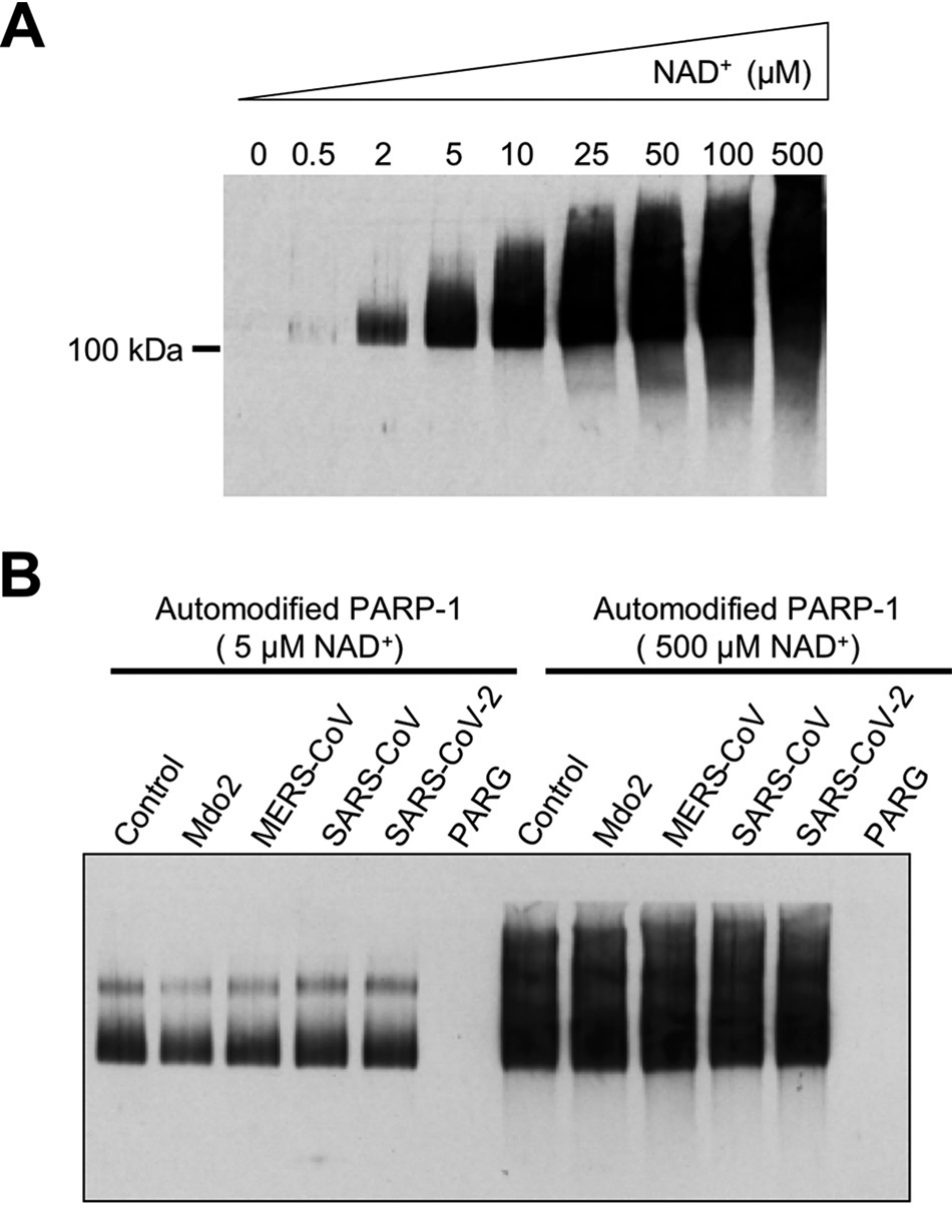
Coronavirus Mac1 proteins do not hydrolyze poly-ADP-ribose (PAR). (A) Differential PARylation of PARP1 by various concentrations of NAD^+^. Recombinant human PARP1 was automodified in a reaction buffer supplemented with increasing concentrations of NAD^+^ to generate substrates for the PAR hydrolase assays. PAR was detected by immunoblot analysis of reaction products with the anti-PAR antibody 96-10. (B) PAR hydrolase assays were performed with PARP1 that was either extensively poly-ADP-ribosylated (500 μM NAD^+^) or partially poly-ADP-ribosylated (5 μM NAD^+^) to produce oligo-ADP-ribose. Macrodomains were incubated with both automodified PARP1 substrates for 1 h. PAR was detected by immunoblotting with the anti-PAR antibody 96-10. PARG (catalytically active 60-kDa fragment) was used as a positive control. The results are representative of 2 independent experiments.

To determine if the CoV Mac1 proteins could remove PAR from proteins, we incubated these proteins with an auto-PARylated PARP1 protein. PARP1 was incubated with increasing concentrations of NAD^+^ to create a range of modification levels (Fig. 10A). We incubated both partially and heavily modified PARP1 with all four macrodomains and poly-ADP-ribose glycohydrolase (PARG) as a positive control for 1 h. While PARG completely removed PAR, none of the macrodomain proteins removed PAR chains from PARP1 (Fig. 10B). We conclude that macrodomain proteins are unable to remove PAR from an automodified PARP1 protein under these conditions.

### ELISAs can be used to measure ADP-ribosylhydrolase activity of macrodomains.

**FIG 11:**
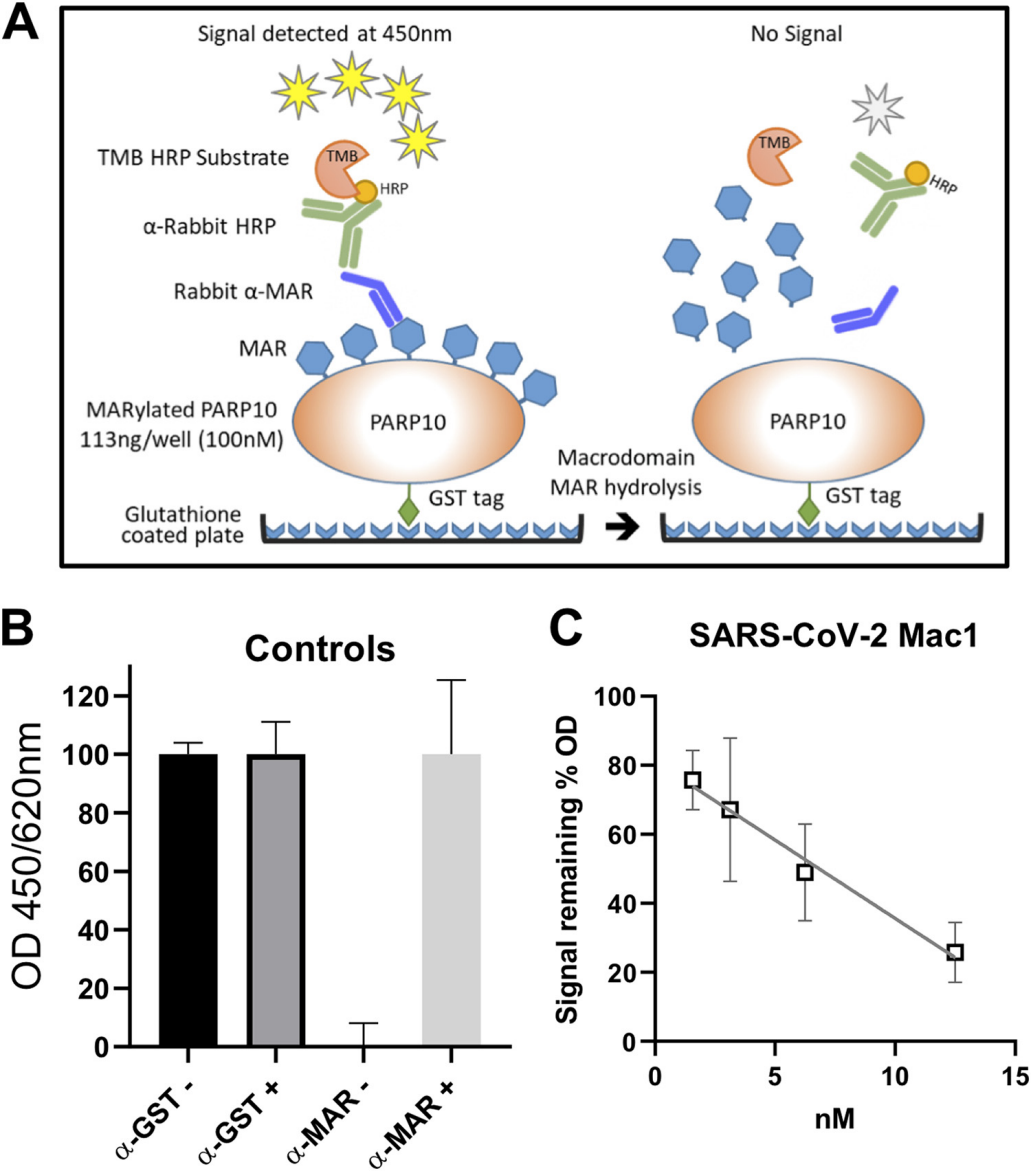
Development of an ELISA to detect de-MARylation. (A) Cartoon schematic of the ELISA. ELISA plates precoated with glutathione and preblocked were used to capture GST-tagged PARP10 proteins, which was used as a substrate for de-MARylation. The removal of MAR was detected by anti-MAR antibodies. (B) MARylated PARP10 (MAR+) and non-MARylated PARP10 (MAR-) with no SARS-CoV-2 Mac1 as controls were detected with anti-mono-ADP-ribose binding reagent (α-MAR) (MAB1076; MilliporeSigma) or with anti-GST (α-GST) (MA4-004; Invitrogen). (C) Starting at 12.5 nM, 2-fold serial dilutions of the SARS-CoV-2 Mac1 protein were incubated in individual wells with MARylated PARP10 CD for 60 min at 37°C. The data are the combined results of 2 independent experiments.

Gel-based assays such as those described above suffer from significant limitations in the number of samples that can be tested at once. A higher-throughput assay will be needed to more thoroughly investigate the activity of these enzymes and to screen for inhibitor compounds. Based on the success of our antibody-based detection of MAR, we developed an enzyme-linked immunosorbent assay (ELISA) that has an ability to detect de-MARylation similar to that of our gel-based assay, but with the ability to do so in a higher-throughput manner (Fig. 11A). First, MARylated PARP10 was added to ELISA plates. Next, the wells were washed and then incubated with different concentrations of the SARS-CoV-2 Mac1 protein for 60 min. After incubation, the wells were washed and treated with anti-MAR binding reagent, followed by horseradish peroxidase (HRP)-conjugated secondary antibody and the detection reagent. As controls, we detected MARylated and non-MARylated PARP10 proteins bound to glutathione plates with anti-GST antibody and anti-MAR binding reagents and their corresponding secondary antibodies (Fig. 11B). SARS-CoV-2 Mac1 was able to remove MAR signal in a dose-dependent manner, and results were plotted to a linear non-regression-fitted line (Fig. 11C). Based on these results, we believe that this ELISA will be a useful tool for screening potential inhibitors of macrodomain proteins.

## DISCUSSION

Here, we report the crystal structure of SARS-CoV-2 Mac1 and its enzyme activity *in vitro*. Structurally, it has a conserved three-layered α/β/α fold typical of the MacroD family of macrodomains and is extremely similar to other CoV Mac1 proteins (Fig. 2 and 6). The conserved CoV macrodomain (Mac1) was initially described as an ADP-ribose-1″-phosphatase (ADRP), as it was shown to be structurally similar to yeast enzymes that have this enzymatic activity (7, 36). Early biochemical studies confirmed this activity for CoV Mac1, though its phosphatase activity for ADP-ribose-1″-phosphate was rather modest (6–8). Later, it was shown that mammalian macrodomain proteins could remove ADP-ribose from protein substrates, indicating protein de-ADP-ribosylation as a more likely function for the viral macrodomains (32, 37, 38). Shortly thereafter, the SARS-CoV, HCoV-229E, feline infectious peritonitis virus (FIPV), several alphavirus, and the hepatitis E virus macrodomains were demonstrated to have de-ADP-ribosylating activity (16–18). However, this activity has not yet been reported for the MERS-CoV or SARS-CoV-2 Mac1 protein.

In this study, we show that the Mac1 proteins from SARS-CoV, MERS-CoV, and SARS-CoV-2 hydrolyze MAR from a protein substrate (Fig. 6). Their enzymatic activities were similar despite sequence divergence of almost 60% between SARS-CoV-2 and MERS-CoV. However, the initial rate associated with the loss of substrate was largest for the SARS-CoV-2 Mac1 protein, particularly under multiple-turnover conditions. It is unclear what structural or sequence differences may account for the increased activity of the SARS-CoV-2 Mac1 protein under these conditions, especially considering the pronounced structural similarities between these proteins, specifically the SARS-CoV Mac1 (0.71 Å RMSD). It is also unclear if these differences would matter in the context of the virus infection, as the relative concentrations of Mac1 and its substrate during infection are not known. We also compared these activities to that of the human Mdo2 macrodomain. Mdo2 had a greater affinity for ADP-ribose than the viral enzymes but had significantly reduced enzyme activity in our experiments. Due to its high affinity for ADP-ribose, it is possible that the Mdo2 protein was partially inhibited by rebinding to the MAR product in these assays. Regardless, these results suggest that the human and viral proteins likely have structural differences that alter their biochemical activities *in vitro*, indicating that it may be possible to create viral macrodomain inhibitors that do not impact the human macrodomains. We also compared the ability of these macrodomain proteins to hydrolyze PAR. None of the macrodomains were able to hydrolyze either partially or heavily modified PARP1, further demonstrating that the primary enzymatic activity of these proteins is to hydrolyze MAR (Fig. 10).

When viral macrodomain sequences are analyzed, it is clear that they have at least 3 highly conserved regions (Fig. 1B) (24). The first region includes the NAAN (residues 37 to 40) and GGG (residues 46 to 48) motifs in the loop between β3 and α2. The second domain includes a GIF (residues 130 to 132) motif in the loop between β6 and α5. The final conserved region is a VGP (residues 96 to 98) motif at the end of β5 and extends into the loop between β5 and α4. Both of the first two domains have well-defined interactions with ADP-ribose (Fig. 3). However, no one has addressed the role of the VGP residues, though our structure indicates that the glycine may interact with a water molecule that makes contact with the β-phosphate. Identifying residues that directly contribute to ADP-ribose binding, hydrolysis, or both by CoV Mac1 proteins will be critical to determining the specific roles of ADP-ribose binding and hydrolysis in CoV replication and pathogenesis.

While all previous studies of macrodomain de-ADP-ribosylation have primarily used radiolabeled substrate, we obtained highly reproducible and robust data utilizing ADP-ribose binding reagents designed to specifically recognize MAR (39, 40). The use of these binding reagents should enhance the feasibility of this assay for many labs that are not equipped for radioactive work. Utilizing these binding reagents, we further developed an ELISA for de-MARylation that has the ability to dramatically increase the number of samples that can be analyzed compared to the gel-based assay. To our knowledge, previously developed ELISAs were used to measure ADP-ribosyltransferase activities (41) but no ELISA has been established to test the ADP-ribosylhydrolase activity of macrodomain proteins. This ELISA should be useful to those in the field to screen compounds for macrodomain inhibitors that could be either valuable research tools or potential therapeutics.

The functional importance of the CoV Mac1 domain has been demonstrated in several reports, mostly utilizing the mutation of a highly conserved asparagine that mediates contact with the distal ribose (Fig. 3B) (18, 21, 22). However, the physiological relevance of Mac1 during SARS-CoV-2 infection has yet to be determined. In addition, the proteins that are targeted by the CoV Mac1 for de-ADP-ribosylation remains unknown. Unfortunately, there are no known compounds that inhibit this domain that could help identify the functions of this protein during infection. The outbreak of COVID-19 has illustrated an urgent need for developing multiple therapeutic drugs targeting conserved coronavirus proteins. Mac1 appears to be an ideal candidate for further drug development based on (i) its highly conserved structure and biochemical activities within CoVs and (ii) its importance for multiple CoVs to cause disease. Targeting Mac1 may also have the benefit of enhancing the innate immune response, as we have shown that Mac1 is required for some CoVs to block IFN production (18, 23). Considering that Mac1 proteins from divergent αCoVs such as HCoV-229E and FIPV also have de-ADP-ribosylating activity (16, 17), it is possible that compounds targeting Mac1 could prevent disease caused by a wide variety of CoVs, including those of veterinary importance like porcine epidemic diarrhea virus (PEDV). Additionally, compounds that inhibit Mac1 in combination with the structure could help identify the mechanisms it uses to bind to its biologically relevant protein substrates, remove ADP-ribose from these proteins, and potentially define the precise function for Mac1 in SARS-CoV-2 replication and pathogenesis. In conclusion, the results described here will be critical for the design and development of highly specific Mac1 inhibitors that could be used therapeutically to mitigate COVID-19 or future CoV outbreaks.

## MATERIALS AND METHODS

### Plasmids.

The SARS-CoV macrodomain (Mac1) (residues 1000 to 1172 of pp1a) was cloned into the pET21a+ expression vector with an N-terminal His tag. The MERS-CoV Mac1 (residues 1110 to 1273 of pp1a) was also cloned into pET21a+ with a C-terminal His tag. SARS-CoV-2 Mac1 (residues 1023 to 1197 of pp1a) was cloned into the pET30a+ expression vector with an N-terminal His tag and a TEV cleavage site (Synbio). The pETM-CN Mdo2 Mac1 (residues 7 to 243) expression vector with an N-terminal His-TEV-V5 tag and the pGEX4T-PARP10-CD (residues 818 to 1025) expression vector with an N-terminal GST tag were previously described (32). All plasmids were confirmed by restriction digestion, PCR, and direct sequencing.

### Protein expression and purification.

A single colony of Escherichia coli cells [C41(DE3)] containing plasmids harboring the constructs of the macrodomain proteins was inoculated into 10 ml LB medium and grown overnight at 37°C with shaking at 250 rpm. The overnight culture was transferred to a shaker flask containing 1 liter 2× Terrific broth (TB) medium at 37°C until the optical density at 600 nm (OD_600_) reached 0.7. The proteins were induced with 0.4 mM IPTG (isopropyl-β-d-thiogalactopyranoside) either at 37°C for 3 h or at 17°C for 20 h. Cells were pelleted at 3,500 × *g* for 10 min and frozen at −80°C. Frozen cells were thawed at room temperature, resuspended in 50 mM Tris (pH 7.6)–150 mM NaCl, and sonicated with the following cycle parameters: amplitude, 50%; pulse length, 30 s; number of pulses, 12, with incubation on ice for >1 min between pulses. The soluble fraction was obtained by centrifuging the cell lysate at 45,450 × *g* for 30 min at 4°C. The expressed soluble proteins were purified by affinity chromatography using a 5-ml prepacked HisTrap HP column on an AKTA Pure protein purification system (GE Healthcare). The fractions were further purified by size exclusion chromatography (SEC) with a Superdex 75 10/300 GL column equilibrated with 20 mM Tris (pH 8.0)–150 mM NaCl, and the protein was sized as a monomer relative to the column calibration standards. To cleave off the His tag from the SARS-CoV-2 Mac1, purified TEV protease was added to purified SARS-CoV-2 Mac1 protein at a ratio of 1:10 (wt/wt) and then passed back through the nickel-nitrilotriacetic acid (Ni-NTA) HP column. Protein was collected in the flowthrough and equilibrated with 20 mM Tris (pH 8.0), 150 mM NaCl. The SARS-CoV-2 Mac1, free from the N-terminal 6-His tag, was used for subsequent crystallization experiments.

For the PARP10 CD protein, the cell pellet was resuspended in 50 mM Tris-HCl (pH 8.0), 500 mM NaCl, 0.1 mM EDTA, 25% glycerol, 1 mM dithiothreitol (DTT) and sonicated as described above. The cell lysate was incubated with 10 ml of glutathione Sepharose 4B resin from GE Healthcare, equilibrated with the same buffer for 2 h, and then applied to a gravity flow column to allow unbound proteins to flow through. The column was washed with the resuspension buffer until the absorbance at 280 nm reached baseline. The bound protein was eluted out of the column with resuspension buffer containing 20 mM reduced glutathione and then dialyzed back into the resuspension buffer overnight at 4°C.

### Isothermal titration calorimetry.

All ITC titrations were performed on a MicroCal PEAQ-ITC instrument (Malvern Pananalytical Inc., MA). All reactions were performed in 20 mM Tris (pH 7.5)–150 mM NaCl using a 100 μM concentration of all macrodomain proteins at 25°C. Titration of 2 mM ADP-ribose or ATP (MilliporeSigma) contained in the stirring syringe included a single 0.4-μl injection, followed by 18 consecutive injections of 2 μl. Data analysis of thermograms was carried out using the “one set of binding sites” model of the MicroCal ITC software to obtain all fitting model parameters for the experiments.

### Differential scanning fluorimetry.

Thermal shift assay with differential scanning fluorimetry (DSF) involved the use of a LightCycler 480 instrument (Roche Diagnostics). In total, a 15-μl mixture containing 8× SYPRO orange (Invitrogen) and 10 μM macrodomain protein in buffer containing 20 mM HEPES–NaOH (pH 7.5) and various concentrations of ADP-ribose were mixed on ice in a 384-well PCR plate (Roche). Fluorescent signals were measured from 25 to 95°C in 0.2°C/30-s steps (excitation, 470 to 505 nm; detection, 540 to 700 nm). The main measurements were carried out in triplicate. Data evaluation and melting temperature (*T_m_*) determination involved use of the Roche LightCycler 480 protein melting analysis software, and data-fitting calculations involved the use of single-site binding curve analysis on GraphPad Prism.

### MAR hydrolase assays.

**(i) Automodification of PARP10 CD protein.** A 10 μM solution of purified PAPR10 CD protein was incubated for 20 min at 37°C with a 1 mM final concentration of β-NAD (β-NAD^+^) (MilliporeSigma) in a reaction buffer (50 mM HEPES, 150 mM NaCl, 0.2 mM DTT, and 0.02% NP-40). MARylated PARP10 was aliquoted and stored at −80°C.

**(ii) PAPR10 CD ADP-ribose hydrolysis.** All reactions were performed at 37°C for the designated time. A 1 μM solution of MARylated PARP10 CD and purified Mac1 protein was added in the reaction buffer (50 mM HEPES, 150 mM NaCl, 0.2 mM DTT, and 0.02% NP-40). The reaction was stopped with addition of 2× Laemmli sample buffer containing 10% β-mercaptoethanol.

Protein samples were heated at 95°C for 5 min before loading and separated onto SDS-PAGE cassettes (Thermo Fisher Scientific Bolt 4 to 12% bis-Tris Plus gels) in MES (morpholineethanesulfonic acid) running buffer. For direct protein detection, the SDS-PAGE gel was stained using InstantBlue protein stain (Expedeon). For immunoblotting, the separated proteins were transferred onto a polyvinylidene difluoride (PVDF) membrane using an iBlot 2 dry blotting system (ThermoFisher Scientific). The blot was blocked with 5% skim milk in phosphate-buffered saline (PBS) containing 0.05% Tween 20 and probed with the anti-mono- or poly-ADP-ribose binding reagents/antibodies MABE1076 (anti-MAR), MABC547 (anti-PAR), and MABE1075 (anti-MAR/PAR) (MilliporeSigma) and the anti-GST tag monoclonal antibody MA4-004 (ThermoFisher Scientific). The primary antibodies were detected with secondary infrared anti-rabbit and anti-mouse immunoglobulin antibodies (LI-COR Biosciences). All immunoblots were visualized using an Odyssey CLx imaging system (LI-COR Biosciences). The images were quantitated using ImageJ (National Institutes for Health [NIH]) or Image Studio software.

**(iii) Kinetic analysis of ADP-ribose hydrolysis.** To quantify the initial rate of substrate decay (*k*) associated with the four macrodomains, each data set represented in the substrate decay immunoblots in Fig. 6C wsas fitted to a decaying exponential with the following functional form: ([S]_initial_ − [S]_final_)*e*^−[^*^k^*^/([S]initial)^*^t^*^]^ + [S]_final_ (Mathematica 12; Wolfram Alpha). The decay plots and resulting values for the fitted parameter *k* along with statistic uncertainty (standard deviations [SD]) are shown in Fig. 6D.

**(iv) ELISA-based MAR hydrolysis.** ELISA Well-Coated glutathione plates (G-Biosciences, USA) were washed with phosphate-buffered saline (PBS) containing 0.05% Tween 20 (PBS-T) and incubated with 50 μl of 100 nM automodified MARylated PARP10 CD in PBS for 1 h at room temperature. Following four washes with PBS-T, various concentrations of SARS-CoV-2 Mac1 were incubated with MARylated PARP10 CD for 60 min at 37°C. Purified macrodomains were 2-fold serially diluted starting at 100 nM in reaction buffer prior to addition to MARylated PARP10 CD. Subsequently, ELISA wells were washed four times with PBS-T and incubated with 50 μl/well of anti-GST (Invitrogen MA4-004) or anti-MAR (MAB1076; MilliporeSigma) diluted 1:5,000 in 5 mg/ml bovine serum albumin (BSA) in PBS-T (BSA5-PBS-T) for 1 h at room temperature. After four additional washes with PBS-T, each well was incubated for 1 h at room temperature with 50 μl of anti-rabbit–HRP (SouthernBiotech, USA) or anti-mouse–HRP (Rockland Immunochemicals, USA) conjugate diluted 1:5,000 in BSA5-PBS-T. The plate was washed four times with PBS-T, and 100 μl of TMB (5,5′-tetramethyl benzidine) peroxidase substrate solution (SouthernBiotech, USA) was added to each well and incubated for 10 min. The peroxidase reaction was stopped with 50 μl per well of 1 M HCl before proceeding to reading. Absorbance was measured at 450 nm and subtracted from 620 nm using a Biotek Powerwave XS plate reader (BioTek). As controls, MARylated PARP10 CD and non-MARylated PARP10 were detected with both anti-MAR and anti-GST antibodies. The absorbance of non-MARylated PARP10 CD detected with anti-MAR antibody was used to establish the background signal. The percentage of signal remaining was calculated by dividing the experimental signal (with enzyme) minus background by the control (no enzyme) minus the background.

### PAR hydrolase assay.

**(i) Automodification of PARP1 protein.** PARP1 was incubated with increasing concentrations of NAD^+^ to generate a range of PARP1 automodification levels. Highly purified human 6-His–PARP1 (42) (5 μg) was incubated for 30 min at 30°C in a reaction buffer containing 100 mM Tris-HCl (pH 8.0), 10 mM MgCl_2_, 10% (vol/vol) glycerol, 10 mM DTT, 0 to 500 μM NAD^+^, 10% (vol/vol) ethanol, and 25 μg/ml calf thymus activated DNA (Sigma-Aldrich).

**(ii) PARP1 ADP-ribose hydrolysis.** To evaluate the PAR hydrolase activity of CoV macrodomains, 200 ng of PARP1 slightly automodified with 5 μM NAD^+^ or highly automodified with 500 μM NAD^+^ was used as the substrate for the de-PARylation assays. Recombinant macrodomain protein (1 μg) was added to the reaction buffer (100 mM Tris-HCl [pH 8.0], 10% [vol/vol] glycerol, and 10 mM DTT) containing automodified PARP1 and incubated for 1 h at 37°C. Recombinant PARG (1 μg) was used as a positive control for PAR erasing (43). Reaction mixtures were resolved on 4 to 12% Criterion XT bis-Tris protein gels, transferred onto nitrocellulose membrane, and probed with the anti-PAR polyclonal antibody 96-10.

### Structure determination.

**(i) Crystallization and data collection.** Purified SARS-CoV-2 Mac1 in 150 mM NaCl–20 mM Tris (pH 8.0) was concentrated to 13.8 mg/ml for crystallization screening. All crystallization experiments were set up using an NT8 drop-setting robot (Formulatrix, Inc.) and UVXPO MRC (Molecular Dimensions) sitting drop vapor diffusion plates at 18°C. One hundred nanoliters of protein and 100 nl crystallization solution were dispensed and equilibrated against 50 μl of the latter. The SARS-CoV-2 Mac1 complex with ADP-ribose was prepared by adding the ligand, from a 100 mM stock in water, to the protein at a final concentration of 2 mM. Crystals were obtained in 1 to 2 days from the Salt Rx HT screen (Hampton Research), condition E10 (1.8 M NaH_2_PO_4_/K_2_HPO_4_, pH 8.2). Refinement screening was conducted using the additive screen HT (Hampton Research) by adding 10% of each additive to the Salt Rx HT E10 condition in a new 96-well UVXPO crystallization plate. The crystals used for data collection were obtained from Salt Rx HT E10 supplemented with 0.1 M NDSB-256 (nondetergent sulfobetaine) from the additive screen. Samples were transferred to a fresh drop composed of 80% crystallization solution and 20% (vol/vol) polyethylene glycol 200 (PEG 200) and stored in liquid nitrogen. X-ray diffraction data were collected at the Advanced Photon Source, IMCA-CAT beamline 17-ID, using a Dectris Eiger 2X 9M pixel array detector.

**(ii) Structure solution and refinement.** Intensities were integrated using XDS (44, 45) via Autoproc (46), and the Laue class analysis and data scaling were performed with Aimless (47). Notably, a pseudotranslational symmetry peak was observed at (0, 0.31 0.5) that was 44.6% of the origin. Structure solution was conducted by molecular replacement with Phaser (48) using a previously determined structure of ADP-ribose bound SARS-CoV-2 Mac1 (PDB 6W02) as the search model. The top solution was obtained in the space group *P*2_1_ with four molecules in the asymmetric unit. Structure refinement and manual model building were conducted with Phenix (49) and Coot (50), respectively. Disordered side chains were truncated to the point for which electron density could be observed. Structure validation was conducted with Molprobity (51), and figures were prepared using the CCP4MG package (52). Superposition of the macrodomain structures was conducted with GESAMT (53).

### Statistical analysis.

All statistical analyses were done using an unpaired two-tailed Student's *t* test to assess differences in mean values between groups, and graphs show means and SD. *P* values of ≤0.05 were considered significant.

### Data availability.

The coordinates and structure factors for SARS-CoV-2 Mac1 were deposited in the Worldwide Protein Databank (wwPDB) with the accession code 6WOJ.

## References

[1] Zhou P, Yang XL, Wang XG, Hu B, Zhang L, Zhang W, Si HR, Zhu Y, Li B, Huang CL, Chen HD, Chen J, Luo Y, Guo H, Jiang RD, Liu MQ, Chen Y, Shen XR, Wang X, Zheng XS, Zhao K, Chen QJ, Deng F, Liu LL, Yan B, Zhan FX, Wang YY, Xiao GF, Shi ZL. 2020. A pneumonia outbreak associated with a new coronavirus of probable bat origin. Nature 579:270–273. doi:10.1038/s41586-020-2012-7. 32015507 PMC7095418

[2] Coronaviridae Study Group of the International Committee on Taxonomy of Viruses. 2020. The species Severe acute respiratory syndrome-related coronavirus: classifying 2019-nCoV and naming it SARS-CoV-2. Nat Microbiol 5:536–544. doi:10.1038/s41564-020-0695-z. 32123347 PMC7095448

[3] Srinivasan S, Cui H, Gao Z, Liu M, Lu S, Mkandawire W, Narykov O, Sun M, Korkin D. 2020. Structural genomics of SARS-CoV-2 indicates evolutionary conserved functional regions of viral proteins. Viruses 12:360. doi:10.3390/v12040360. PMC7232164 32218151

[4] Wu C, Liu Y, Yang Y, Zhang P, Zhong W, Wang Y, Wang Q, Xu Y, Li M, Li X, Zheng M, Chen L, Li H. 2020. Analysis of therapeutic targets for SARS-CoV-2 and discovery of potential drugs by computational methods. Acta Pharm Sin B 10:766–788. doi:10.1016/j.apsb.2020.02.008. 32292689 PMC7102550

[5] Fehr AR, Perlman S. 2015. Coronaviruses: an overview of their replication and pathogenesis, Methods Mol Biol 1282:1–23. doi:10.1007/978-1-4939-2438-7_1. 25720466 PMC4369385

[6] Egloff MP, Malet H, Putics A, Heinonen M, Dutartre H, Frangeul A, Gruez A, Campanacci V, Cambillau C, Ziebuhr J, Ahola T, Canard B. 2006. Structural and functional basis for ADP-ribose and poly(ADP-ribose) binding by viral macro domains. J Virol 80:8493–8502. doi:10.1128/JVI.00713-06. 16912299 PMC1563857

[7] Putics A, Filipowicz W, Hall J, Gorbalenya AE, Ziebuhr J. 2005. ADP-ribose-1″-monophosphatase: a conserved coronavirus enzyme that is dispensable for viral replication in tissue culture. J Virol 79:12721–12731. doi:10.1128/JVI.79.20.12721-12731.2005. 16188975 PMC1235854

[8] Saikatendu KS, Joseph JS, Subramanian V, Clayton T, Griffith M, Moy K, Velasquez J, Neuman BW, Buchmeier MJ, Stevens RC, Kuhn P. 2005. Structural basis of severe acute respiratory syndrome coronavirus ADP-ribose-1″-phosphate dephosphorylation by a conserved domain of nsP3. Structure 13:1665–1675. doi:10.1016/j.str.2005.07.022. 16271890 PMC7126892

[9] Cho CC, Lin MH, Chuang CY, Hsu CH. 2016. Macro domain from Middle East respiratory syndrome coronavirus (MERS-CoV) is an efficient ADP-ribose binding module: crystal structure and biochemical studies. J Biol Chem 291:4894–4902. doi:10.1074/jbc.M115.700542. 26740631 PMC4777827

[10] Xu Y, Cong L, Chen C, Wei L, Zhao Q, Xu X, Ma Y, Bartlam M, Rao Z. 2009. Crystal structures of two coronavirus ADP-ribose-1″-monophosphatases and their complexes with ADP-ribose: a systematic structural analysis of the viral ADRP domain. J Virol 83:1083–1092. doi:10.1128/JVI.01862-08. 18987156 PMC2612350

[11] Tan J, Vonrhein C, Smart OS, Bricogne G, Bollati M, Kusov Y, Hansen G, Mesters JR, Schmidt CL, Hilgenfeld R. 2009. The SARS-unique domain (SUD) of SARS coronavirus contains two macrodomains that bind G-quadruplexes. PLoS Pathog 5:e1000428. doi:10.1371/journal.ppat.1000428. 19436709 PMC2674928

[12] Chatterjee A, Johnson MA, Serrano P, Pedrini B, Joseph JS, Neuman BW, Saikatendu K, Buchmeier MJ, Kuhn P, Wuthrich K. 2009. Nuclear magnetic resonance structure shows that the severe acute respiratory syndrome coronavirus-unique domain contains a macrodomain fold. J Virol 83:1823–1836. doi:10.1128/JVI.01781-08. 19052085 PMC2643772

[13] Makrynitsa GI, Ntonti D, Marousis KD, Birkou M, Matsoukas MT, Asami S, Bentrop D, Papageorgiou N, Canard B, Coutard B, Spyroulias GA. 2019. Conformational plasticity of the VEEV macro domain is important for binding of ADP-ribose. J Struct Biol 206:119–127. doi:10.1016/j.jsb.2019.02.008. 30825649 PMC7111667

[14] Malet H, Coutard B, Jamal S, Dutartre H, Papageorgiou N, Neuvonen M, Ahola T, Forrester N, Gould EA, Lafitte D, Ferron F, Lescar J, Gorbalenya AE, de Lamballerie X, Canard B. 2009. The crystal structures of Chikungunya and Venezuelan equine encephalitis virus nsP3 macro domains define a conserved adenosine binding pocket. J Virol 83:6534–6545. doi:10.1128/JVI.00189-09. 19386706 PMC2698539

[15] Rack JG, Perina D, Ahel I. 2016. Macrodomains: structure, function, evolution, and catalytic activities. Annu Rev Biochem 85:431–454. doi:10.1146/annurev-biochem-060815-014935. 26844395

[16] Li C, Debing Y, Jankevicius G, Neyts J, Ahel I, Coutard B, Canard B. 2016. Viral macro domains reverse protein ADP-ribosylation. J Virol 90:8478–8486. doi:10.1128/JVI.00705-16. 27440879 PMC5021415

[17] Eckei L, Krieg S, Butepage M, Lehmann A, Gross A, Lippok B, Grimm AR, Kummerer BM, Rossetti G, Luscher B, Verheugd P. 2017. The conserved macrodomains of the non-structural proteins of Chikungunya virus and other pathogenic positive strand RNA viruses function as mono-ADP-ribosylhydrolases. Sci Rep 7:41746. doi:10.1038/srep41746. 28150709 PMC5288732

[18] Fehr AR, Channappanavar R, Jankevicius G, Fett C, Zhao J, Athmer J, Meyerholz DK, Ahel I, Perlman S. 2016. The conserved coronavirus macrodomain promotes virulence and suppresses the innate immune response during severe acute respiratory syndrome coronavirus infection. mBio 7:e01721-16. doi:10.1128/mBio.01721-16. 27965448 PMC5156301

[19] Kim DS, Challa S, Jones A, Kraus WL. 2020. PARPs and ADP-ribosylation in RNA biology: from RNA expression and processing to protein translation and proteostasis. Genes Dev 34:302–320. doi:10.1101/gad.334433.119. 32029452 PMC7050490

[20] Fehr AR, Singh SA, Kerr CM, Mukai S, Higashi H, Aikawa M. 2020. The impact of PARPs and ADP-ribosylation on inflammation and host-pathogen interactions. Genes Dev 34:341–359. doi:10.1101/gad.334425.119. 32029454 PMC7050484

[21] Eriksson KK, Cervantes-Barragán L, Ludewig B, Thiel V. 2008. Mouse hepatitis virus liver pathology is dependent on ADP-ribose-1″-phosphatase, a viral function conserved in the alpha-like supergroup. J Virol 82:12325–12334. doi:10.1128/JVI.02082-08. 18922871 PMC2593347

[22] Fehr AR, Athmer J, Channappanavar R, Phillips JM, Meyerholz DK, Perlman S. 2015. The nsp3 macrodomain promotes virulence in mice with coronavirus-induced encephalitis. J Virol 89:1523–1536. doi:10.1128/JVI.02596-14. 25428866 PMC4300739

[23] Grunewald ME, Chen Y, Kuny C, Maejima T, Lease R, Ferraris D, Aikawa M, Sullivan CS, Perlman S, Fehr AR. 2019. The coronavirus macrodomain is required to prevent PARP-mediated inhibition of virus replication and enhancement of IFN expression. PLoS Pathog 15:e1007756. doi:10.1371/journal.ppat.1007756. 31095648 PMC6521996

[24] Alhammad YMO, Fehr AR. 2020. The viral macrodomain counters host antiviral ADP-ribosylation. Viruses 12:384. doi:10.3390/v12040384. PMC7232374 32244383

[25] Abraham R, Hauer D, McPherson RL, Utt A, Kirby IT, Cohen MS, Merits A, Leung AKL, Griffin DE. 2018. ADP-ribosyl-binding and hydrolase activities of the alphavirus nsP3 macrodomain are critical for initiation of virus replication. Proc Natl Acad Sci U S A 115:E10457–E10466. doi:10.1073/pnas.1812130115. 30322911 PMC6217424

[26] Abraham R, McPherson RL, Dasovich M, Badiee M, Leung AKL, Griffin DE. 2020. Both ADP-ribosyl-binding and hydrolase activities of the alphavirus nsP3 macrodomain affect neurovirulence in mice. mBio 11:e03253-19. doi:10.1128/mBio.03253-19. 32047134 PMC7018654

[27] McPherson RL, Abraham R, Sreekumar E, Ong SE, Cheng SJ, Baxter VK, Kistemaker HA, Filippov DV, Griffin DE, Leung AK. 2017. ADP-ribosylhydrolase activity of Chikungunya virus macrodomain is critical for virus replication and virulence. Proc Natl Acad Sci U S A 114:1666–1671. doi:10.1073/pnas.1621485114. 28143925 PMC5321000

[28] Parvez MK. 2015. The hepatitis E virus ORF1 'X-domain' residues form a putative macrodomain protein/Appr-1″-pase catalytic-site, critical for viral RNA replication. Gene 566:47–53. doi:10.1016/j.gene.2015.04.026. 25870943 PMC7127128

[29] Vuksanovic N, Silvaggi NR. 2020. 6WEY: high-resolution structure of the SARS-CoV-2 NSP3 Macro X domain. Protein Data Bank. doi:10.2210/pdb6WEY/pdb.

[30] Michalska K, Kim Y, Jedrzejczak R, Maltseva NI, Stols L, Endres M, Joachimiak A. 2020. Crystal structures of SARS-CoV-2 ADP-ribose phosphatase: from the apo form to ligand complexes. IUCrJ 7:814–824. doi:10.1107/S2052252520009653. PMC7467174 32939273

[31] Frick DN, Virdi RS, Vuksanovic N, Dahal N, Silvaggi NR. 2020. Molecular basis for ADP-ribose binding to the Mac1 domain of SARS-CoV-2 nsp3. Biochemistry (Mosc) 59:2608–2615. doi:10.1021/acs.biochem.0c00309. PMC7341687 32578982

[32] Jankevicius G, Hassler M, Golia B, Rybin V, Zacharias M, Timinszky G, Ladurner AG. 2013. A family of macrodomain proteins reverses cellular mono-ADP-ribosylation. Nat Struct Mol Biol 20:508–514. doi:10.1038/nsmb.2523. 23474712 PMC7097781

[33] Leung AKL, McPherson RL, Griffin DE. 2018. Macrodomain ADP-ribosylhydrolase and the pathogenesis of infectious diseases. PLoS Pathog 14:e1006864. doi:10.1371/journal.ppat.1006864. 29566066 PMC5864081

[34] Lei J, Kusov Y, Hilgenfeld R. 2018. Nsp3 of coronaviruses: structures and functions of a large multi-domain protein. Antiviral Res 149:58–74. doi:10.1016/j.antiviral.2017.11.001. 29128390 PMC7113668

[35] Heer CD, Sanderson DJ, Voth LS, Alhammad YMO, Schmidt MS, Trammell SAJ, Perlman S, Cohen MS, Fehr AR, Brenner C. 2020. Coronavirus infection and PARP expression dysregulate the NAD metabolome: an actionable component of innate immunity. J Biol Chem doi:10.1074/jbc.RA120.015138. PMC7834058 33051211

[36] Shull NP, Spinelli SL, Phizicky EM. 2005. A highly specific phosphatase that acts on ADP-ribose 1″-phosphate, a metabolite of tRNA splicing in Saccharomyces cerevisiae. Nucleic Acids Res 33:650–660. doi:10.1093/nar/gki211. 15684411 PMC548356

[37] Rosenthal F, Feijs KL, Frugier E, Bonalli M, Forst AH, Imhof R, Winkler HC, Fischer D, Caflisch A, Hassa PO, Luscher B, Hottiger MO. 2013. Macrodomain-containing proteins are new mono-ADP-ribosylhydrolases. Nat Struct Mol Biol 20:502–507. doi:10.1038/nsmb.2521. 23474714

[38] Sharifi R, Morra R, Appel CD, Tallis M, Chioza B, Jankevicius G, Simpson MA, Matic I, Ozkan E, Golia B, Schellenberg MJ, Weston R, Williams JG, Rossi MN, Galehdari H, Krahn J, Wan A, Trembath RC, Crosby AH, Ahel D, Hay R, Ladurner AG, Timinszky G, Williams RS, Ahel I. 2013. Deficiency of terminal ADP-ribose protein glycohydrolase TARG1/C6orf130 in neurodegenerative disease. EMBO J 32:1225–1237. doi:10.1038/emboj.2013.51. 23481255 PMC3642678

[39] Gibson BA, Conrad LB, Huang D, Kraus WL. 2017. Generation and characterization of recombinant antibody-like ADP-ribose binding proteins. Biochemistry (Mosc) 56:6305–6316. doi:10.1021/acs.biochem.7b00670. PMC6465537 29053245

[40] Affar EB, Duriez PJ, Shah RG, Winstall E, Germain M, Boucher C, Bourassa S, Kirkland JB, Poirier GG. 1999. Immunological determination and size characterization of poly(ADP-ribose) synthesized in vitro and in vivo. Biochim Biophys Acta 1428:137–146. doi:10.1016/S0304-4165(99)00054-9. 10434031

[41] Asokanathan C, Tierney S, Ball CR, Buckle G, Day A, Tanley S, Bristow A, Markey K, Xing D, Yuen CT. 2018. An ELISA method to estimate the mono ADP-ribosyltransferase activities: e.g in pertussis toxin and vaccines. Anal Biochem 540–541:15–19. doi:10.1016/j.ab.2017.10.025. 29108883

[42] Langelier MF, Planck JL, Servent KM, Pascal JM. 2011. Purification of human PARP-1 and PARP-1 domains from Escherichia coli for structural and biochemical analysis. Methods Mol Biol 780:209–226. doi:10.1007/978-1-61779-270-0_13. 21870263

[43] Wang Z, Gagne JP, Poirier GG, Xu W. 2014. Crystallographic and biochemical analysis of the mouse poly(ADP-ribose) glycohydrolase. PLoS One 9:e86010. doi:10.1371/journal.pone.0086010. 24465839 PMC3897571

[44] Kabsch W. 1988. Evaluation of single-crystal X-ray diffraction data from a position-sensitive detector. J Appl Crystallogr 21:916–924. doi:10.1107/S0021889888007903.

[45] Kabsch W. 2010. Xds. Acta Crystallogr D Biol Crystallogr 66:125–132. doi:10.1107/S0907444909047337. 20124692 PMC2815665

[46] Vonrhein C, Flensburg C, Keller P, Sharff A, Smart O, Paciorek W, Womack T, Bricogne G. 2011. Data processing and analysis with the autoPROC toolbox. Acta Crystallogr D Biol Crystallogr 67:293–302. doi:10.1107/S0907444911007773. 21460447 PMC3069744

[47] Evans PR. 2011. An introduction to data reduction: space-group determination, scaling and intensity statistics. Acta Crystallogr D Biol Crystallogr 67:282–292. doi:10.1107/S090744491003982X. 21460446 PMC3069743

[48] McCoy AJ, Grosse-Kunstleve RW, Adams PD, Winn MD, Storoni LC, Read RJ. 2007. Phaser crystallographic software. J Appl Crystallogr 40:658–674. doi:10.1107/S0021889807021206. 19461840 PMC2483472

[49] Adams PD, Afonine PV, Bunkoczi G, Chen VB, Davis IW, Echols N, Headd JJ, Hung LW, Kapral GJ, Grosse-Kunstleve RW, McCoy AJ, Moriarty NW, Oeffner R, Read RJ, Richardson DC, Richardson JS, Terwilliger TC, Zwart PH. 2010. PHENIX: a comprehensive Python-based system for macromolecular structure solution. Acta Crystallogr D Biol Crystallogr 66:213–221. doi:10.1107/S0907444909052925. 20124702 PMC2815670

[50] Emsley P, Lohkamp B, Scott WG, Cowtan K. 2010. Features and development of Coot. Acta Crystallogr D Biol Crystallogr 66:486–501. doi:10.1107/S0907444910007493. 20383002 PMC2852313

[51] Chen VB, Arendall WB, III, Headd JJ, Keedy DA, Immormino RM, Kapral GJ, Murray LW, Richardson JS, Richardson DC. 2010. MolProbity: all-atom structure validation for macromolecular crystallography. Acta Crystallogr D Biol Crystallogr 66:12–21. doi:10.1107/S0907444909042073. 20057044 PMC2803126

[52] Potterton L, McNicholas S, Krissinel E, Gruber J, Cowtan K, Emsley P, Murshudov GN, Cohen S, Perrakis A, Noble M. 2004. Developments in the CCP4 molecular-graphics project. Acta Crystallogr D Biol Crystallogr 60:2288–2294. doi:10.1107/S0907444904023716. 15572783

[53] Krissinel E. 2012. Enhanced fold recognition using efficient short fragment clustering. J Mol Biochem 1:76–85. 27882309 PMC5117261

[54] Evans P. 2006. Scaling and assessment of data quality. Acta Crystallogr D Biol Crystallogr 62:72–82. doi:10.1107/S0907444905036693. 16369096

[55] Diederichs K, Karplus PA. 1997. Improved R-factors for diffraction data analysis in macromolecular crystallography. Nat Struct Biol 4:269–275. doi:10.1038/nsb0497-269. 9095194

[56] Weiss MS. 2001. Global indicators of X-ray data quality. J Appl Cryst 34:130–135. doi:10.1107/S0021889800018227.

[57] Evans P. 2012. Resolving some old problems in protein crystallography. Science 336:986–987. doi:10.1126/science.1222162. 22628641

[58] Karplus PA, Diederichs K. 2012. Linking crystallographic model and data quality. Science 336:1030–1033. doi:10.1126/science.1218231. 22628654 PMC3457925

